# Asthma and its relationship to mitochondrial copy number: Results from the Asthma Translational Genomics Collaborative (ATGC) of the Trans-Omics for Precision Medicine (TOPMed) program

**DOI:** 10.1371/journal.pone.0242364

**Published:** 2020-11-25

**Authors:** Maxwell P. Cocco, Evan White, Shujie Xiao, Donglei Hu, Angel Mak, Patrick Sleiman, Mao Yang, Kevin R. Bobbitt, Hongsheng Gui, Albert M. Levin, Samantha Hochstadt, Kyle Whitehouse, Dean Rynkowski, Andrea J. Barczak, Gonçalo Abecasis, Thomas W. Blackwell, Hyun Min Kang, Deborah A. Nickerson, Soren Germer, Jun Ding, David E. Lanfear, Frank Gilliland, W. James Gauderman, Rajesh Kumar, David J. Erle, Fernando Martinez, Hakon Hakonarson, Esteban G. Burchard, L. Keoki Williams

**Affiliations:** 1 Center for Individualized and Genomic Medicine Research (CIGMA), Department of Internal Medicine, Henry Ford Health System, Detroit, Michigan, United States of America; 2 Department of Medicine, University of California San Francisco, San Francisco, California, United States of America; 3 Center for Applied Genomics, Children's Hospital of Philadelphia, Philadelphia, Pennsylvania, United States of America; 4 Department of Pediatrics, Perelman School of Medicine, University of Pennsylvania, Philadelphia, Pennsylvania, United States of America; 5 Department of Public Health Sciences, Henry Ford Health System, Detroit, Michigan, United States of America; 6 Lung Biology Center and UCSF CoLabs, University of California San Francisco, San Francisco, California, United States of America; 7 Center for Statistical Genetics, University of Michigan, Ann Arbor, Michigan, United States of America; 8 Regeneron Pharmaceuticals, Inc., Tarrytown, New York, United States of America; 9 Department of Genome Sciences, University of Washington, Seattle, Washington, United States of America; 10 Northwest Genomics Center, Seattle, Washington, United States of America; 11 Brotman Baty Institute, Seattle, Washington, United States of America; 12 New York Genome Center, New York, New York, United States of America; 13 Human Statistical Genetics Unit, Laboratory of Genetics and Genomics, National Institute on Aging, National Institutes of Health, Bethesda, Maryland, United States of America; 14 Department of Preventive Medicine, Keck School of Medicine, University of Southern California, Los Angeles, California, United States of America; 15 Department of Pediatrics, Northwestern University Feinberg School of Medicine, Chicago, Illinois, United States of America; 16 Arizona Respiratory Center and Department of Pediatrics, University of Arizona, Tucson, Arizona, United States of America; 17 Department of Bioengineering & Therapeutic Sciences, University of California San Francisco, San Francisco, California, United States of America; Cincinnati Children's Hospital Medical Center, UNITED STATES

## Abstract

**Background:**

Mitochondria support critical cellular functions, such as energy production through oxidative phosphorylation, regulation of reactive oxygen species, apoptosis, and calcium homeostasis.

**Objective:**

Given the heightened level of cellular activity in patients with asthma, we sought to determine whether mitochondrial DNA (mtDNA) copy number measured in peripheral blood differed between individuals with and without asthma.

**Methods:**

Whole genome sequence data was generated as part of the Trans-Omics for Precision Medicine (TOPMed) Program on participants from the Study of Asthma Phenotypes and Pharmacogenomic Interactions by Race-ethnicity (SAPPHIRE) and the Study of African Americans, Asthma, Genes, & Environment II (SAGE II). We restricted our analysis to individuals who self-identified as African American (3,651 asthma cases and 1,344 controls). Mitochondrial copy number was estimated using the sequencing read depth ratio for the mitochondrial and nuclear genomes. Respiratory complex expression was assessed using RNA-sequencing.

**Results:**

Average mitochondrial copy number was significantly higher among individuals with asthma when compared with controls (SAPPHIRE: 218.60 vs. 200.47, P<0.001; SAGE II: 235.99 vs. 223.07, P<0.001). Asthma status was significantly associated with mitochondrial copy number after accounting for potential explanatory variables, such as participant age, sex, leukocyte counts, and mitochondrial haplogroup. Despite the consistent relationship between asthma status and mitochondrial copy number, the latter was not associated with time-to-exacerbation or patient-reported asthma control. Mitochondrial respiratory complex gene expression was disproportionately lower in individuals with asthma when compared with individuals without asthma and other protein-encoding genes.

**Conclusions:**

We observed a robust association between asthma and higher mitochondrial copy number. Asthma having an effect on mitochondria function was also supported by lower respiratory complex gene expression in this group.

## Introduction

Asthma is a chronic disease of the respiratory system often characterized by airway inflammation, excess mucus production, and bronchial constriction [1]. In the United States, it is currently estimated that 11 million adults and 11 million children suffer from asthma [2,3].

Mitochondria are subcellular organelles that produce up to 90% of cellular energy through the citric acid cycle and oxidative phosphorylation [4]. In addition to producing adenosine triphosphate, mitochondria are involved in other critical intracellular processes such maintaining calcium homeostasis, triggering apoptotic cell death, and regulating potentially toxic levels of reactive oxygen species (ROS) [5–8].

Since mitochondria possess their own genome [9], it is possible to estimate the number of mitochondrial genomes per cell (i.e., mitochondrial copy number) by obtaining the mitochondrial DNA (mtDNA) to nuclear DNA (nDNA) ratio. Previous studies have shown that average mitochondrial copy numbers in white blood cells (WBCs) differ for individuals with respiratory diseases, such as chronic obstructive pulmonary disease (COPD) when compared to healthy individuals [10,11], but to our knowledge such studies have not been performed among individuals with asthma alone. *In vitro* and animal models suggest that various environmental exposures, including aeroallergens, can damage electron transport chain (ETC) respiratory complex proteins, promote ROS production, and increase oxidative stress [12,13]. Decreased expression of ETC genes in bronchial epithelial cells may predispose to allergic inflammation and increase airway responsiveness following allergen challenge [12]. Oxidative stress has also been associated with mitochondrial copy number [14,15]. Therefore, differences in mitochondrial copy number and ETC gene expression among individuals with asthma may identify subtle forms of mitochondrial dysfunction contributing to disease. Such findings and insights may ultimately improve asthma phenotyping and treatment.

In this study, we evaluated whether mitochondrial copy numbers in blood differed among individuals with and without asthma in participants from both the Study of Asthma Phenotypes and Pharmacogenomic Interactions by Race-ethnicity (SAPPHIRE) cohort and the Study of African Americans, Asthma, Genes & Environment (SAGE II). We also consider potential causes mtDNA copy number differences by measuring expression of genes involved in oxidative phosphorylation and by assessing mitochondrial volume and membrane potential.

## Methods

### Patients and settings

Both the SAPPHIRE and SAGE II cohorts and the research presented here were approved by the Institutional Review Boards at Henry Ford Health System and the University of California San Francisco. Both cohorts are part of the Asthma Translational Genomics Collaborative (ATGC) in National Heart Lung and Blood Institute’s (NHLBI) Trans-Omics for Precision Medicine (TOPMed) program. Written informed consent was obtained from all participants or their guardians, as well as written assent from participants <18 years of age, prior to study enrollment and the collection of participant data.

All individuals in the SAPPHIRE cohort were patients from a single health system serving metropolitan Detroit and southeast Michigan. This study has been described in detail elsewhere [16]. Briefly, eligible asthma case individuals had to be 12–56 years of age at the time of recruitment, have a previously documented clinical diagnosis of asthma from a physician, and have no prior history of chronic obstructive pulmonary disease or congestive heart failure. Control individuals were recruited from the same patient population and met the same entry requirements with the exception of not having a prior diagnosis of asthma. Since whole genome sequencing (WGS) was primarily performed in African American participants in SAPPHIRE as part of the TOPMed program, we restricted our analysis to African American participants and to individuals who were ≥18 years of age at the time of enrollment.

At the time of enrollment, SAPPHIRE participants underwent a detailed clinical evaluation which included a detailed questionnaire, anthropomorphic measurements, lung function testing, and specimen collection. Level of asthma control was evaluated using the Asthma Control Test (ACT), a self-reported instrument consisting of 5 questions, each scored on a 5-point Likert scale [17]. Composite ACT scores <20 and ≥20 are considered uncontrolled asthma and controlled asthma, respectively. Lung function testing was performed in accordance with 2005 ATS/ERS guidelines [18]. Complete blood counts with 5-part white blood cell (WBC) differential were measured in blood collected in ethylenediaminetetraacetic acid tubes using a UniCel DxH 800 Coulter Cellular Analysis System (Beckman Coulter, Inc., Brea, CA).

The SAGE II cohort consisted of African American individuals with and without asthma recruited from clinics throughout the San Francisco Bay Area. As a criterion for enrollment, subjects had to self-identify as African American and report that both biological parents and all biological grandparents identified themselves as African American. Participants were between the ages of 8 and 21 years at the time of enrollment. Cases had a history of physician-diagnosed asthma and two or more asthma symptoms (wheezing, coughing, or shortness of breath) in the preceding two years. Participants underwent a detailed evaluation which included obtaining anthropomorphic measurements, testing lung function in accordance with 2005 ATS/ERS guidelines [18], completing a detailed survey with questions on medication use and smoking status, and collecting blood for various laboratory tests (e.g., blood counts). Growth charts (available at http://www.cdc.gov/nccdphp/dnpao/growthcharts/resources/sas.htm) were used to calculate the BMI percentile of SAGE II participants [19]. Only SAGE II participants <20 years of age were included in the current analysis.

### Whole genome sequencing, mitochondrial copy number, mitochondrial haplogroup, and genetic ancestry

DNA was isolated from whole blood, and samples of sufficient quantity and quality were sent to the University of Washington’s Northwest Genomic Center or the New York Genome Center for sequencing. Pre-sequencing quality checks included an assessment of DNA quantity and sex discordance. Libraries for WGS were created using Illumina’s TruSeq DNA PCR-Free Library Preparation Kit. Sequencing was performed on the Illumina HiSeq X platform with paired-end 150bp reads. The Illumina package, bcl2fastq v2 15.0, was used to generate individual FASTQ files. The alignment pipeline can be found at https://github.com/CCDG/Pipeline-Standardization/blob/master/PipelineStandard.md. In brief, the TOPMed Data Coordinating Center at the Northwest Genomics Center aligned the raw sequence reads in FASTQ format to the human reference genome to create binary sequence alignment (BAM) files using the Burrows-Wheeler Aligner algorithm, BWA-MEM [20]. The alignment files were then sent to the Informatics Research Center at the University of Michigan where joint variant calls were performed. We first used existing array data to identify and remove discordant samples based on genotype mismatches. Samples with >10% genotype missingness or sex mismatched X chromosome heterozygosity were also excluded. The R package GENESIS was used to assess for relatedness and population structure [21]. In the case of monozygotic twins, one from each pair was randomly selected and removed from the analysis. Individuals with African ancestry >5 standard deviations [SD] below the mean (~20% global African ancestry) and individuals with closer than 3^rd^ degree relationship with other study participants were excluded as a post hoc sensitivity analysis. We used the BAM files to estimate mitochondrial copy number based on the ratio of read depth using the fastMitoCalc program included in the mitoAnalyzer software package [22]. As a check of the mitochondrial copy number estimates derived using WGS data, we agnostically selected a set of 50 blood samples to quantitate copy number using both the real-time polymerase chain reaction (RT-PCR)-based method described by Ajaz *et al*. [23] and sequence read depth. The primers selected for this RT-PCR assay identified unique and singular portions of the mitochondrial and nuclear genomes (i.e., to avoid mitochondrial pseudogene amplification and dilutional effects from copy number differences in the nuclear genome) [24]. Mitochondrial copy number estimates were significantly lower using PCR-based method when compared with the read depth method (67.6 ± 24.4 standard deviation [SD] vs. 232.3 ± 77.3, respectively; P = 2.2x10^-16^ [paired T-test]). However, the measurements obtained by both methods were strongly correlated (Pearson’s r = 0.74, P = 5.9 x 10^−10^) as shown in Supplementary S1 Fig, suggesting that *relative* copy number estimates between individuals were similar using both approaches.

BAM files containing mitochondrial read alignments were separated using SAMtools v1.8 software. The pipeline for mitochondrial haplogroup assignments involved the use of Cloudgene (locally installed) with mtDNA-Server v1.1.3 and the program HaploGrep 2 [25,26]. Global ancestry (i.e., the overall proportion of continental group ancestry) was estimated using the programs RFMix and Admixture [27,28].

### Medication use and asthma severity

A subset of the SAPPHIRE participants with asthma had detailed records on medication fills as a result of their health plan membership; these data included pharmacy claims from health plan and retail pharmacies. We estimated inhaled corticosteroid (ICS) use in the 6 months prior to the visit date using algorithms that we had developed and reported on previously [29,30]. Average daily exposure to short acting beta agonist (SABA) medication was estimated using records of SABA fills in the 3-month period preceding study enrollment. Separate metrics of SABA use were calculated for nebulizer use and metered dose inhaler (MDI) use, as we have previously shown that both the form and the frequency of SABA medication use may be related to asthma severity [31,32]. Average daily SABA use for each participant was estimated by summing the total number of doses (e.g., 50 puffs per canister) dispensed for that individual in the 91 days preceding enrollment and dividing by 91.

We also estimated asthma severity using a previously described metric which uses both SABA and oral corticosteroid prescription fills in the preceding year [33]. The lowest severity group, Group 1, had no oral corticosteroid (OCS) fills and ≤1 SABA fill in the 12 months prior to the time of evaluation. Group 3 had one of the following: 2 OCS fills, >6 SABA fills, or the combination of 1 OCS fill and ≥4 SABA fills. Group 4 had either ≥3 OCS fills or the combination of 2 OCS fills and >6 SABA fills. Group 2 encompassed all other combinations of SABA and OCS fills in the year prior to the study visit.

### RNA sequence (RNA-seq) data

Blood was collected from SAPPHIRE participants at the time of enrollment for later RNA analysis. Blood was collected and stored in PAXgene Blood RNA tubes (BD Biosciences, San Jose, CA) at -80°C until the time of analysis. The TruSeq Stranded Total RNA Library Prep Kit with Ribo-Zero Globin (Illumina, San Diego, CA) was used to create sequencing libraries on a random sample of African American SAPPHIRE participants with and without asthma. Libraries were sequenced on an Illumina HiSeq. FastQC v0.11.5 (available at http://www.bioinformatics.babraham.ac.uk/projects/fastqc) and MultiQC v0.8 were used to assess RNA-seq quality, including the read length distribution and depth [34]. The program BBDuk (BBMap v36.49) was used to trim reads and remove Illumina adapter sequence (available at https://sourceforge.net/projects/bbmap/). The software program HISAT2 2.1.0 was used to map reads to human genome build GRCh38.p5 [35], and mapped reads were quantified at the transcript level using StringTie v1.3.3 [36,37]. RSeQC (v2.6.4) was used to provide post-alignment QC, including the distribution of mapped reads, coverage uniformity, strand specificity after alignment [38]. Samples that did not pass any of the above QC measures were excluded from the analysis, we also excluded samples from batches that didn’t include both cases and control participants. We restricted our assessment to mitochondrial and nuclear genes encoding proteins making up ETC complexes. These complexes compose the final critical pathway for oxidative metabolism of foodstuffs; complexes I-IV participate in the transfer of electrons and the creation of a proton potential across the inner mitochondrial membrane, and this gradient drives the formation of cellular energy as ATP by complex V [39]. Gene expression was quantified as transcripts per kilobase per million reads (TPM); this measure normalizes read counts for a gene by accounting for its length (in kilobases) and the total number of reads for a sample (in millions). In addition, the R-package, DESeq2, was used to normalize read counts across individuals and all protein-encoding genes in order to assess for differences in expression by asthma status [40].

### Mitochondrial assessment in peripheral blood leukocytes by flow cytometry

We invited a subset of SAPPHIRE participants to provide a new blood sample for mitochondrial assessment by flow cytometry. This group included individuals with asthma and a high mitochondrial copy number (>300) (eligible n = 61), and individuals with (eligible n = 119) and without (eligible n = 107) asthma who had a mitochondrial copy number near the average for their group (± 10 from the mean). We evaluated the first to respond from these eligible groups—4 (average copy number 371.3 ± 25 SD), 4 (average copy number 217.9 ± 1.3 SD), and 5 (average copy number 198.1 ± 5.7 SD), respectively. Approximately 12 milliliters (ml) of blood were collected from each participant into heparinized tubes; blood was centrifuged with 14 ml of Ficoll-Paque Plus (GE Healthcare Life Sciences) to isolate WBCs. After centrifugation, both the buffy coat layer and the granulocyte layer were removed. The latter was combined with 20 ml of FACS lysing solution (BD Biosciences); the sample was then mixed and incubated for 30 minutes. The peripheral blood mononuclear cells (PBMCs) from the buffy coat were centrifuged, and the resulting cell pellet was washed multiple times and resuspended in sterile 15 ml of 1x phosphate-buffered saline solution. A 10 microliter (μl) sample from each cell suspension was stained with trypan blue, and the cells were counted manually using a hemacytometer. We used MitoTracker Red CMXRos (Molecular Probes, Inc., Eugene, OR) to quantify mitochondria–dye accumulation was dependent on both mitochondrial number and membrane potential. Following the manufacture’s guidelines, cells were incubated for 30 minutes with prewarmed staining solution containing a 100 nanomolar concentration of MitoTracker Red CMXRos. Cells were then fixed with buffer containing 4% formaldehyde and stored at -80°C until the time of analysis. For the flow cytometry analysis, granulocytes were stained with 2.5 μl of anti-CD203c antibody conjugated with allophycocyanin (APC) dye (BioLegend, San Diego, CA), 3 μl of anti-CD117 antibody conjugated with APC-Cy7^TM^ dye (BioLegend), 3 μl of anti-Siglec 8 antibody conjugated with phycoerythrin (PE) dye (BioLegend), and 5 μl of anti-CD45 antibody conjugated with Brilliant Violet 421^TM^ dye (BioLegend); PBMCs were stained with 5 μl of anti-CD45 antibody conjugated with Brilliant Violet 421^TM^ dye (BioLegend), 2 μl of anti-CD3 antibody conjugated with APC-Cy7^TM^ dye (BioLegend), 5 μl of anti-CD4 antibody conjugated with peridinin-chlorophyll-protein-Cy5.5 dye (BD Biosciences), and 3 μl of anti-CD56 antibody conjugated with PE-CF594 dye (BioLegend). Eosinophils (CD45+, Siglec 8+, and CD203c-), T-helper cells (CD45+, CD3+, and CD4+), and natural killer (NK) cells (CD45+, CD3-, and CD56+) were assessed for MitoTracker staining intensity using a LSRFortessa flow cytometer (BD Biosciences). Compensation beads (BD CompBead, BD Biosciences) were used to correct for spectral overlap between fluorochrome-labeled antibodies, and the gates for positively stained cells were positioned using “fluorescence minus one” controls. Leukocyte populations were identified using the light scatter characteristics of live cells in the forward scatter and side scatter channels to collect 100,000 gated events. Flow cytometry data were analyzed using FacsDiva v8.0.1 software (BD Biosciences).

### Statistical analysis

The primary study aim was to assess the relationship between mitochondrial copy number (outcome) and asthma status (predictor variable). Secondary analyses included assessing factors associated with mitochondrial copy number (outcome) among individuals with asthma and, conversely, assessing whether mitochondrial copy number (predictor variable) was separately associated with the outcomes of asthma status, exacerbations, and control.

Differences in baseline (i.e., at the time of the initial study visit) characteristics between individuals with and without asthma were assessed using chi-squared tests and t-tests for categorical and continuous variables, respectively. We then used Welch’s two-sample t-test to compare copy number differences between mitochondrial haplogroups categorized by location of origin. We compared copy numbers between individuals with either African mitochondrial haplogroups (L0, L1, L2, L3, and L4) or east Eurasian haplogroups combined (B, F, A, D, E, and C/Z) with west Eurasian haplogroups combined (U/K [U2, U3, U4, U5, U7, and K], J/T, HV/H/V, W, and I); individuals with west Eurasian haplogroups were the reference group for these comparisons. The above population distribution of haplogroup assignments has been described elsewhere [4,41,42]. We also compared copy numbers between individuals with African mitochondrial haplogroups (L0, L1, L2, L3, and L4), M haplogroups (D, E, C/Z, M7, and other M), and N + R haplogroups (N macrohaplogroups [X, A, W, and I] and the R sub-macrohaplogroups [U/K, B, F, HV/H/V, and J/T]); in these comparisons, individuals with N + R haplogroups were considered the reference group. Haplogroup categories were also stratified by asthma status in order to assess within haplogroup differences in mitochondrial copy numbers by asthma status. The R package, metafor, was used to meta-analyze the standardized mean difference in mitochondrial copy number between asthma case and control participants from the SAPPHIRE and SAGE II cohorts [43].

To understand the factors contributing to mitochondrial copy number, we used linear regression to model copy number as a function of patient asthma status, patient age, sex (male = 0, female = 1), proportion of African ancestry, body mass index (BMI) in kilograms per meter squared, smoking status (past or never smoker = 0, active smoker = 1), percent of predicted forced expiratory volume at 1 second (FEV_1_), absolute WBC counts for neutrophils, monocytes, lymphocytes, and eosinophils (basophils were not included given their scant number), platelet count, and mitochondrial haplogroup (included using dummy variables for African haplogroups [L0, L1, L2, and L3] and combined east Eurasian haplogroups as compared with the combined west Eurasian haplogroups). Given the small number of individuals with the L4 haplogroup, we did not include them in these analyses. Since most SAGE II participants were children, BMI percentile was used in lieu of BMI [19]. Only a small subset of SAGE II participants had WBC counts, so we performed analyses with and without this variable.

In order to identify potential factors within individuals with asthma contributing to higher mitochondrial copy number, we restricted the model to SAPPHIRE participants with asthma. In this analysis, we included variables related to asthma treatment and severity, such as the composite ACT score, asthma severity score, SABA exposure, and ICS exposure. These particular variables were only available for SAPPHIRE participants.

Logistic regression was used to model asthma status (dependent variable) as a function of mitochondrial copy number with other potential explanatory and confounding variables, such as patient age, sex, proportion of African ancestry, BMI, smoking status, percent of predicted FEV_1_, absolute WBC counts for neutrophils, monocytes, lymphocytes, and eosinophils (basophils were not included given their scant number), and mitochondrial haplogroups.

As a sensitivity analysis, we reassessed the relationship between asthma status and mitochondrial copy number after removing individuals with a low proportion of African ancestry or those who were closely related to other study participants. Specifically, we removed 23 individuals from SAPPHIRE and 1 individual from SAGE II whose African ancestry was >5 SD below the mean (i.e., a global African ancestry proportion of approximately 20% or less), and we excluded 203 individuals from SAPPIRE and 0 individuals from SAGE II whose kinship suggested closer than a 3^rd^ degree relationship (coefficient of relationship >0.125) with another study participant.

To evaluate whether mitochondrial copy number was associated with asthma complications and control, we used a Cox proportional hazards regression to prospectively model the time to severe asthma exacerbations and we used logistic regression to model baseline asthma control (ACT score <20 [uncontrolled] vs. ≥20 [controlled]). Prospective data were only available in SAPPHIRE participants. Severe asthma exacerbations were defined as those requiring oral corticosteroid burst treatment, an emergency department visit, or hospitalization. The survival analysis modeled the time between study enrollment and the first severe exacerbation. Individuals were censored on their last recorded clinical visit or at the time of disenrollment from the health plan.

The non-parametric Mann–Whitney U test was used for the univariate comparison of gene expression TPM values between asthma cases and controls. DESeq2 was used to assess for differential gene expression by asthma status while also adjusting for patient age, sex, global proportion of African ancestry, and absolute cells counts for neutrophils, monocytes, lymphocytes, eosinophils, and platelets. We also used surrogate variable analysis (SVA) implemented in the package svaseq to account for hidden batch effects; 10 surrogate variables were used to adjust the models [44]. The Fisher’s exact test was used to compare the expression distribution (i.e., number of higher, lower, and not-significantly different expressed genes for individuals with asthma as compared with individuals without asthma) between ETC genes and other protein-encoding genes (restricted to genes with >10 total read counts across all individuals).

The t-test was used to compare mean MitoTracker intensities in blood leukocyte populations (i.e., eosinophils, T-helper cells, and NK cells) between individuals with asthma and high mitochondrial copy number vs. controls with average mitochondrial number, as well as between individuals with asthma and average mitochondrial copy number vs. controls with average mitochondrial number. All analyses were performed using R statistical software [45]. Unless otherwise specified, a p-value <0.05 was considered statistically significant.

## Results

**Fig 1:**
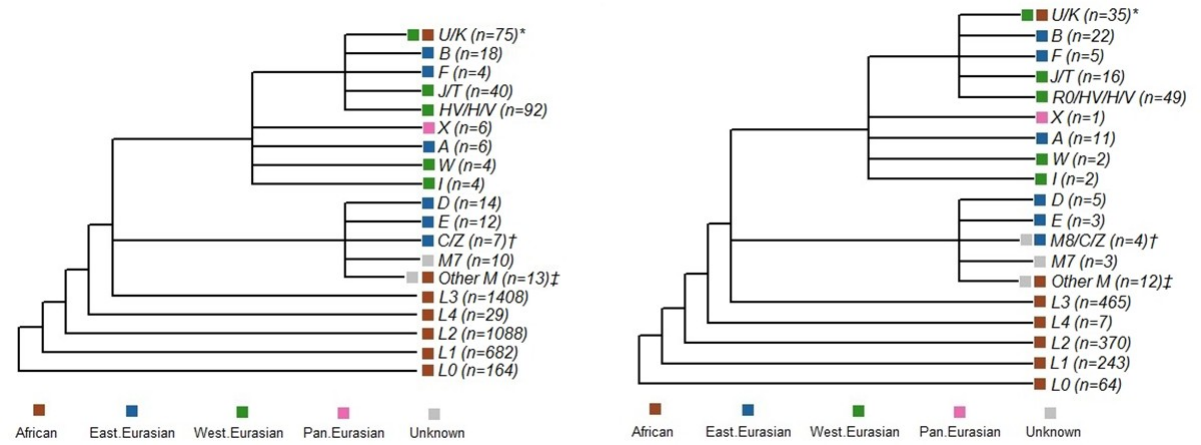
Mitochondrial haplogroup distributions. Shown are the mitochondrial haplogroup distributions of the 3,676 SAPPHIRE participants (**A**) and the 1,319 SAGE II participants (**B**). Branching shows haplogroup derivation and color coding denotes the geographic identity of the haplogroup. *In SAPPHIRE this group consisted of 34 individuals with an African haplogroup (U6) and 41 with a west Eurasian haplogroup (U2, U3, U4, U5, U7, and K), and in SAGE II this group consisted of 16 individuals with an African haplogroup (U6) and 19 with a west Eurasian haplogroup (U4, U5, U8, and K). †In SAPPHIRE this group consisted of 7 individuals with an east Eurasian haplogroup (C, Z), and in SAGE II this group consisted of 3 individuals with an east Eurasian haplogroup (C, Z) and 1 individual with a haplogroup of unknown origin (M8). ‡In SAPPHIRE this group consisted of 2 individuals with an African haplogroup (M1) and 11 individuals with a haplogroup of unknown origin (M2, M23, M30, and M32), and in SAGE II this group consisted of 6 individuals with an African haplogroup (M1) and 6 individuals with a haplogroup of unknown origin (M3, M5, M6, and M23).

The characteristics of participants used in this analysis are shown in Table 1. All individuals were African American by self-report. The SAPPHIRE cohort analytic set consisted of 3,676 individuals (2,828 cases and 848 controls), and SAGE II set consisted of 1,319 individuals (823 cases and 496 controls). While SAPPHIRE participants were older at the time of enrollment (≥18 years) when compared with SAGE II participants (<20 years), both groups were similar in their proportion of African ancestry (~80% in both cohorts) and both had a similar mitochondrial haplogroup distribution. Approximately, 92%, 91%, 87%, 88% of SAPPHIRE cases, SAPPHIRE controls, SAGE II cases, and SAGE II controls, respectively, had mitochondrial haplogroups of African origin (i.e., haplogroups L0, L1, L2, L3, and L4). A detailed breakdown of mitochondrial haplogroup distribution for each cohort can be seen in Fig 1A and 1B.

**Table 1:** Characteristics of African American participants from the SAPPHIRE and SAGE II cohorts stratified by asthma status*.

Variable	SAPPHIRE cohort			SAGE II cohort		
	Participants with asthma (n = 2,828)	Participants without asthma (n = 848)	P-value†	Participants with asthma (n = 823)	Participants without asthma (n = 496)	P-value†
Age (years)–mean ± SD	36.98 ± 12.12	39.60 ± 12.55	<0.001	14.06 ± 3.69	15.85 ± 3.71	< 0.001
Female–no. (%)	1955 (69.1)	581 (68.5)	0.766	407 (49.5)	284 (57.3)	0.007
African ancestry (%)–mean ± SD‡	0.80 ± 0.12	0.81 ± 0.12	0.226	0.79 ± 0.13	0.78 ± 0.12	0.431
BMI (kg/m^2^)–mean ± SD	33.17 ± 9.39	31.75 ± 7.89	<0.001	--	--	--
BMI percentile—mean ± SD§	--	--	--	76.39 ± 24.74	70.95 ± 27.21	0.005
Smoking status–no. (%)	--	--	<0.001	--	--	0.001
Current	765 (27.1)	107 (12.6)	--	68 (8.3)	68 (13.7)	--
Past	322 (11.4)	98 (11.6)	--	1 (0.1)	2 (0.4)	
Never	1740 (61.5)	643 (75.8)	--	753 (91.6)	426 (85.9)	--
Percent of predicted FEV_1_ –mean ± SD||	85.14 ± 19.67	96.70 ± 15.29	<0.001	99.01 ± 13.79	101.64 ± 9.22	< 0.001
Composite ACT score <20 –no. (%)¶	1,580 (55.9)	--	--	--	--	--
Asthma severity score–mean ± SD**	1.68 ± 0.85	--	--	--	--	--
ICS medication use at baseline–no. (%)††	237 (46.3)	--	--	282 (58.51)	--	--
Mitochondrial copy number–mean ± SD‡‡	218.60 ± 58.80	200.47 ± 64.95	<0.001	235.99 ± 59.22	223.07 ± 61.48	<0.001
WBC counts (1000/μL)–mean ± SD	6.68 ± 2.32	6.10 ± 1.80	<0.001	6.22 ± 1.98	6.20 ± 2.12	0.951
Neutrophil counts	3.70 ± 1.92	3.24 ± 1.33	<0.001	--	--	--
Monocyte counts	0.46 ± 0.17	0.43 ± 0.15	0.006	--	--	--
Lymphocyte counts	2.28 ± 0.80	2.26 ± 0.72	0.633	--	--	--
Eosinophil counts	0.21 ± 0.19	0.14 ± 0.11	<0.001	--	--	--
Hemoglobin (g/dL)–mean ± SD	13.29 ± 1.55	13.24 ± 1.40	0.492	--	--	--
Platelet count (1000/μL)–mean ± SD	242.35 ± 67.00	242.30 ± 61.09	0.985	--	--	--
Mitochondrial haplogroup—no. (%)§§	--	--	0.429	--	--	0.735
L0	122 (4.3)	42 (5.0)	--	42 (5.1)	22 (4.4)	--
L1	516 (18.2)	166 (19.6)	--	149 (18.1)	94 (19.0)	--
L2	826 (29.2)	262 (30.9)	--	225 (27.3)	145 (29.2)	--
L3	1110 (39.2)	298 (35.1)	--	291 (35.4)	174 (35.1)	--
L4	22 (0.8)	7 (0.8)	--	5 (0.6)	2 (0.4)	--
M	45 (1.6)	11 (1.3)	--	17 (2.1)	10 (2.0)	--
N + R	187 (6.6)	62 (7.3)	--	94 (11.4)	49 (9.9)	--
East Eurasian	48 (1.7)	13 (1.5)	--	32 (3.9)	17 (3.4)	--
West Eurasian	141 (5.0)	40 (4.7)	--	61 (7.4)	27 (5.4)	--

SAPPHIRE denotes Study of Asthma Phenotypes and Pharmacogenomic Interactions by Race-ethnicity; SAGE II, Study of African Americans, Asthma, Genes, & Environment II; SD, standard deviation; BMI, body mass index; FEV_1_, forced expiratory volume at 1 second; ACT, asthma control test; ICS, inhaled corticosteroid; and WBC, white blood cell.

*The SAPPHIRE study sample was restricted to participants aged ≥18 years at enrollment and the SAGE II study samples was restricted to participants aged <20 years at enrollment.

†P-value for the difference between study participants with and without asthma in each cohort.

‡The average proportion of African ancestry among African Americans was estimated using a set of autosomal markers which spanned the nuclear genome.

§Growth charts specific for age and sex (available at http://www.cdc.gov/nccdphp/dnpao/growthcharts/resources/sas.htm) were used to calculate the BMI percentile of SAGE II participants.

||Based on the predictive equations from Hankinson *et al*. (Am J Respir Crit Care Med. 1999 Jan;159[1]:179–87).

¶Composite ACT scores <20 are considered “uncontrolled” asthma.

**This scoring algorithm, which is based on asthma rescue medication use, was developed by Allen-Ramey *et al*. (J Manag Care Pharm 2006; 12:310–21). This measure was calculated on the 512 individuals with asthma and available pharmacy claims information.

††This measure was calculated on the 512 individuals with asthma and available pharmacy claims information in SAPPHIRE participants and was based on cross-sectional self-report of use in SAGE II participants.

‡‡The mitochondrial copy number estimate was for whole blood. It was based on the sequencing read depth ratio between mitochondrial and nuclear DNA isolated from blood leukocytes.

§§Number of study individuals with a given mitochondrial haplogroup. As shown in Fig 1, the M haplogroups consist of D, E, C/Z, M7 and other M; the N macrohaplogroups consist of X, A, W, I, and the R sub-macrohaplogroups; and R sub-macrohaplogroups consist of U/K, B, F, HV/H/V, and J/T. Geographical mitochondrial haplogroup assignments were based on those described by Pereira *et al*. (Am J Hum Genet. 2009; 84:628–40). Haplogroups L0, L1, L2, L3, and L4 are considered to be African; haplogroups B, F, A, D, E, and C/Z are considered to be East Eurasian; and haplogroups U/K, J/T, HV/H/V, I, and W are considered to be West Eurasian.

Asthma cases and controls also differed in consistent ways in both cohorts, such as case participants were significantly younger, had greater a BMI (greater BMI percentile in SAGE II), and lower percent of predicted FEV_1_ when compared with their respective control group. Sex distribution and smoking patterns differed between cohorts (cases were more likely to have smoked in SAPPHIRE but less likely to have smoked in SAGE II). In SAPPHIRE participants, asthma cases had significantly greater cell counts when compared with controls: total WBC counts (6,680 cells/μL vs. 6,100 cells/μL), neutrophil counts (3,700 cells/μL vs. 3,240 cells/μL), monocyte counts (460 cells/μL vs. 430 cells/μL), and eosinophil counts (210 cells/μL vs. 140 cells/μL). In both SAPPHIRE and SAGE II, mitochondrial copy numbers were significantly higher among individuals with asthma when compared with those without asthma (SAPPHIRE: 218.60 vs. 200.47, P<0.001; SAGE II: 235.99 vs. 223.07, P<0.001).

Table 2 evaluates the relationship between mitochondrial haplogroup and mitochondrial copy number. Among SAPPHIRE participants, individuals with west Eurasian mitochondrial haplogroups had lower average mitochondrial counts than did individuals with L1, L3, and east Eurasian haplogroups. SAPPHIRE individuals with other African haplogroups (L0 and L2) and SAGE II individuals did not have significantly different mitochondrial copy numbers when compared with individuals with west Eurasian haplogroups. Defining haplogroup clusters based on the branching shown in Fig 1 (as opposed to geographical haplogroup assignments) demonstrated essentially the same results (Supplementary S1 Table), since the majority of individuals in the N + R haplogroup branch had haplogroups of west Eurasian origin (181 [72.7%] of 249 in SAPPHIRE and 88 [61.5%] of 143 in SAGE II).

**Table 2:** Relationship between mitochondria haplogroup and copy number among African American participants in the SAPPHIRE and SAGE II cohorts*.

Mitochondrial haplogroup†	SAPPHIRE cohort				SAGE II cohort			
	Number of participants‡	African ancestry (mean ± SD)§	Copy number (mean ± SD)||	P-value¶	Number of participants‡	African ancestry (mean ± SD)§	Copy number (mean ± SD)||	P-value¶
L0	164	0.81 ± 0.09	201.95 ± 59.25	0.827	64	0.82 **±** 0.09	228.63 **±** 59.02	0.734
L1	682	0.82 ± 0.09	215.91 ± 60.95	0.010	243	0.80 **±** 0.11	228.75 **±** 52.67	0.618
L2	1088	0.82 ± 0.09	211.52 ± 58.99	0.079	370	0.81 **±** 0.10	228.66 **±** 60.10	0.621
L3	1408	0.82 ± 0.09	218.27 ± 62.84	0.001	465	0.81 **±** 0.09	237.46 **±** 65.52	0.063
East Eurasian	61	0.77 ± 0.16	222.52 ± 44.71	0.008	49	0.67 **±** 0.18	222.18 **±** 56.82	0.739
West Eurasian	181	0.56 ± 0.27	203.33 ± 57.60	Reference	88	0.58 **±** 0.18	225.48 **±** 52.70	Reference

SAPPHIRE denotes the Study of Asthma Phenotypes and Pharmacogenomic Interactions by Race-ethnicity; SAGE II, Study of African Americans, Asthma, Genes, & Environment II; and SD, standard deviation.

*The SAPPHIRE study sample was restricted to participants aged ≥18 years at enrollment and the SAGE II study samples was restricted to participants aged <20 years at enrollment.

†Geographical mitochondrial haplogroup assignments were based on those described by Pereira *et al*. (Am J Hum Genet. 2009; 84:628–40). Haplogroups L0, L1, L2, and L3 are considered to be African. Given the small number of study individuals with non-African haplogroups, we grouped most of the remaining haplogroups into the broad categories of East Eurasian and West Eurasian.

‡Includes individuals with and without asthma in each cohort.

§African ancestry was estimated using a set of autosomal markers which spanned the nuclear genome.

||The mitochondrial copy number estimate was for whole blood. It was based on the sequencing read depth ratio between mitochondrial and nuclear DNA isolated from blood leukocytes.

¶P-values were calculated using the Welch two sample t-test to compare mitochondrial copy numbers between West Eurasian haplogroups (referent) and the other haplogroups.

Within haplogroup categories, average mitochondrial copy numbers were consistently higher among individuals with asthma when compared with those without asthma (Table 3). These differences were statistically significant for L1, L2, L3, and west Eurasian haplogroups in SAPPHIRE and the L2 haplogroup in SAGE II. Defining haplogroup clusters based on the branching shown in Fig 1 (as opposed to geographical haplogroup assignments) demonstrated essentially the same results with the exception of the small number of individuals with M haplogroups who showed a non-significant relationship in the opposite direction (Supplementary S2 Table). There were too few individuals with the L4 haplogroup for comparison in SAGE II, so this group was dropped from all subsequent analyses.

**Table 3:** Relationship between mitochondria haplogroup and copy number among African American participants in the SAPPHIRE and SAGE II cohorts stratified by asthma status*.

Mitochondrial haplogroup†	SAPPHIRE cohort					SAGE II cohort					Meta-analysis		
	Participants with asthma		Participants without asthma		P-value§	Participants with asthma		Participants without asthma		P-value§	Standardized difference (95% CI)	Heterogeneity P-value||	P-value¶
	No.	Mitochondria copy number (mean ± SD)‡	No.	Mitochondria copy number (mean ± SD) ‡		No.	Mitochondria copy number (mean ± SD) ‡	No.	Mitochondria copy number (mean ± SD) ‡				
L0	122	204.66 ± 53.87	42	194.09 ± 72.85	0.392	42	232.17 **±** 49.65	22	221.87 **±** 74.60	0.564	0.18 (-0.11–0.47)	0.988	0.233
L1	516	220.56 ± 60.50	166	201.44 ± 60.22	<0.001	149	232.97 **±** 48.13	94	222.06 **±** 58.82	0.133	0.28 (0.14–0.43)	0.496	<0.001
L2	826	216.40 ± 56.96	262	196.14 ± 62.65	<0.001	225	237.64 **±** 59.24	145	214.73 **±** 58.95	<0.001	0.36 (0.24–0.48)	0.754	<0.001
L3	1110	221.65 ± 60.33	298	205.67 ± 70.11	<0.001	291	240.19 **±** 66.28	174	232.89 **±** 64.14	0.242	0.21 (0.10–0.32)	0.214	<0.001
East Eurasian	48	222.98 ± 44.89	13	220.80 ± 45.79	0.880	32	227.06 **±** 57.03	17	212.99 **±** 56.99	0.417	0.15 (-0.27–0.58)	0.648	0.485
West Eurasian	141	208.98 ± 56.32	40	183.41 ± 58.31	0.016	61	227.25 **±** 55.11	27	221.47 **±** 47.55	0.619	0.32 (0.04–0.60)	0.245	0.024

SAPPHIRE denotes the Study of Asthma Phenotypes and Pharmacogenomic Interactions by Race-ethnicity; SAGE II, Study of African Americans, Asthma, Genes, & Environment II; SD, standard deviation; and CI, confidence interval.

*The SAPPHIRE study sample was restricted to participants aged ≥18 years at enrollment and the SAGE II study samples was restricted to participants aged <20 years at enrollment.

†Geographical mitochondrial haplogroup assignments were based on those described by Pereira *et al*. (Am J Hum Genet. 2009; 84:628–40). Haplogroups L0, L1, L2, and L3 are considered to be African. Given the small number of study individuals with non-African haplogroups, we grouped most of the remaining haplogroups into the broad categories of East Eurasian and West Eurasian.

‡The mitochondrial copy number estimate was for whole blood. It was based on the sequencing read depth ratio between mitochondrial and nuclear DNA isolated from blood leukocytes.

§P-values were calculated using the Welch two sample t-test to compare mitochondrial copy numbers between individuals with and without asthma.

||Assessment of the difference in P-values from the SAPPHIRE and SAGE II cohorts. A P-value<0.05 would signify a statistically significant difference in the P-values between cohorts.

¶Meta-analysis P-value for the standardized difference in mitochondrial copy number between individuals within and without asthma for the SAPPHIRE and SAGE II cohorts combined.

Effect estimates for the factors associated with mitochondrial copy number were remarkably consistent between the SAPPHIRE and SAGE II cohorts (Tables 4 and 5, respectively). Asthma was consistently and significantly associated with greater mitochondrial copy numbers in all models tested for both cohorts. Excluding individuals whose African ancestry was >5 SD below the mean or who had a coefficient of relationship >0.125 among SAPPHIRE participants did not substantively affect the significant relationship between asthma status and mitochondrial copy number (Supplementary S3 Table). Similarly, excluding the one SAGE II with African ancestry >5 SD below the mean also did not affect the results (data not shown). White blood cell counts were inversely associated with mitochondrial copy number in both groups (also shown in Supplementary S4 Table for SAGE II). In the SAPPHIRE cohort, all leukocyte counts were *inversely* associated with mitochondrial copy number, and all but monocytes were significantly associated with copy number in the multivariable analysis (Table 4). Platelet counts were *positively* associated with mitochondrial copy number. The relationship between cell counts by cell type and mitochondrial copy number stratified by asthma status is shown in Supplementary S2 Fig. Significant interactions by asthma status were seen for neutrophils (P = 2.68x10^-4^) and platelets (P = 0.009); however, asthma was consistently associated with higher mitochondrial counts regardless of cell type count (i.e., a cross-over interaction was not observed for any cell type).

**Table 4:** Factors associated with mitochondrial copy number among African American SAPPHIRE participants.

Variable	Univariable Analysis			Model 1†		Model 2‡		Model 3§		Model 4||	
	R^2^*	Unadjusted parameter estimate	P-value	Adjusted parameter estimate	P-value	Adjusted parameter estimate	P-value	Adjusted parameter estimate	P-value	Adjusted parameter estimate	P-value
Asthma status	0.016	18.13	<0.001	19.42	<0.001	34.19	<0.001	18.19	<0.001	33.25	<0.001
Age (years)	0.003	-0.28	<0.001	-0.17	0.039	--	--	--	--	-0.20	0.096
Female Sex	<0.001	0.17	0.937	1.68	0.447	--	--	--	--	-6.54	0.048
African Ancestry	0.003	29.64	<0.001	33.97	<0.001	--	--	--	--	9.44	0.483
BMI (kg/m^2^)	0.002	-0.33	0.003	-0.39	<0.001	--	--	--	--	0.05	0.790
Smoking status	<0.001	0.36	0.879	-2.93	0.219	--	--	--	--	7.00	0.058
Percent of predicted FEV_1_	<0.001	-0.04	0.452	0.05	0.383	--	--	--	--	-0.09	0.253
Total WBC count	0.133	-11.70	<0.001	--	--	--	--	--	--	--	--
Neutrophils	0.120	-13.79	<0.001	--	--	-14.58	<0.001	--	--	-14.36	<0.001
Monocytes	0.043	-87.33	<0.001	--	--	-13.84	0.148	--	--	-18.32	0.067
Lymphocytes	0.033	-16.62	<0.001	--	--	-13.14	<0.001	--	--	-12.67	<0.001
Eosinophils	0.003	-21.91	0.013	--	--	-20.21	0.014	--	--	-24.34	0.004
Platelet count	0.008	0.10	<0.001	--	--	0.217	<0.001	--	--	0.23	<0.001
Mitochondrial haplogroup	0.005	--	--	--	--	--	--	--	--	--	--
L0 vs West Eurasian	--	-1.38	0.833	--	--	--	--	-0.74	0.909	-1.47	0.875
L1 vs West Eurasian	--	12.58	0.014	--	--	--	--	12.98	0.010	9.47	0.218
L2 vs West Eurasian	--	8.19	0.094	--	--	--	--	8.55	0.078	6.38	0.390
L3 vs West Eurasian	--	14.94	0.002	--	--	--	--	14.77	0.002	13.21	0.073

SAPPHIRE denotes Study of Asthma Phenotypes and Pharmacogenomic Interactions by Race-ethnicity; BMI, body mass index; FEV_1_, forced expiratory volume at 1 second; and WBC, white blood count.

*The coefficient of determination (R^2^) represented the percent of the variation in the outcome variable (i.e., mitochondrial copy number) that could be accounted for by each variable in the univariable analyses.

†Model 1 assessed the relationship between mitochondrial copy number in blood leukocytes (dependent variable) and asthma status (main explanatory variable). This model adjusted for patient age in years, sex (female = 1, male = 0), proportion of African ancestry, BMI, smoking status (past or never smoker = 0, active smoker = 1), and percent of predicted FEV_1_. Complete data were available for 3,675 individuals in Model 1, which had an adjusted R^2^ = 0.024.

‡Model 2 assessed the relationship between mitochondrial copy number in blood leukocytes (dependent variable) and asthma status (main explanatory variable). This model adjusted for absolute white blood cell counts and platelet counts (in increments of 1000 cells per microliter). Complete data were available for 2,031 individuals in Model 2, which had an adjusted R^2^ = 0.219.

§Model 3 assessed the relationship between mitochondrial copy number in blood leukocytes (dependent variable) and asthma status (main explanatory variable). This model adjusted for mitochondrial haplogroup, and only individuals with the L0, L1, L2, L3, and West Eurasian haplogroups were included. Complete data were available for 3,523 individuals in Model 3, which had an adjusted R^2^ = 0.020.

||Model 4 assessed the relationship between mitochondrial copy number in blood leukocytes (dependent variable) and asthma status (main explanatory variable); this model included all of the variables from models 1–3. Complete data were available for 1,942 individuals in Model 4, which had an adjusted R^2^ = 0.229.

**Table 5:** Factors associated with mitochondrial copy number among African American SAGE II participants.

Variable	Univariable Analysis			Model 1†		Model 2‡		Model 3§	
	R^2^*	Unadjusted parameter estimate	P-value	Adjusted parameter estimate	P-value	Adjusted parameter estimate	P-value	Adjusted parameter estimate	P-value
Asthma status	0.010	12.91	<0.001	28.31	<0.001	12.86	<0.001	27.62	<0.001
Age (years)	0.033	-2.94	<0.001	-2.43	<0.001	--	--	-2.28	<0.001
Female sex	0.001	-5.52	0.097	-5.12	0.145	--	--	-6.54	0.072
African ancestry proportion	0.001	17.17	0.190	32.25	0.023	--	--	36.18	0.039
BMI percentile	0.001	0.07	0.312	0.01	0.852	--	--	0.0076	0.916
Smoking status	<0.001	-27.19	0.436	-46.66	0.237	--	--	-45.15	0.251
Percent of predicted FEV_1_	0.003	-0.30	0.024	-0.14	0.294	--	--	-0.18	0.201
Total WBC count	0.243	-11.11	<0.001	--	--	--	--	--	--
Mitochondrial haplogroup	0.002	--	--	--	--	--	--	--	--
L0 vs West Eurasian	--	3.15	0.750	--	--	3.63	0.713	-11.84	0.285
L1 vs West Eurasian	--	3.28	0.663	--	--	4.31	0.565	-7.48	0.389
L2 vs West Eurasian	--	3.19	0.656	--	--	4.28	0.548	-2.65	0.750
L3 vs West Eurasian	--	11.98	0.088	--	--	12.85	0.066	0.013	0.999

SAGE II denotes the Study of African Americans, Asthma, Genes, & Environment II; BMI, body mass index; FEV_1_, forced expiratory volume at 1 second; and WBC, white blood count.

*The coefficient of determination (R^2^) represented the percent of the variation in the outcome variable (i.e., mitochondrial copy number) that could be accounted for by each variable in the univariable analyses.

†Multivariable linear regression model 1 (Model 1) assessed the relationship between asthma and overall mitochondrial copy number in blood. This model adjusted for patient age in years, sex (female = 1, male = 0), proportion of African ancestry per individual (continuous), BMI percentile (continuous), smoking status (past or never smoker = 0, active smoker = 1), and percent of predicted FEV_1_ (continuous). Complete data were available for 1017 individuals in Model 1, which had an adjusted R^2^ = 0.087.

‡Multivariable linear regression model 2 (Model 2) assessed the relationship between overall mitochondrial copy number in blood and asthma status as well as mitochondrial haplogroup. Haplogroup was included as categorical variable with West Eurasian as reference level. Only cases with haplogroup L0, L1, L2, L3 and West Eurasian were included in the model. Complete data were available for 1230 individuals in Model 2, which had an adjusted R^2^ = 0.012.

§Multivariable linear regression model 3 (Model 3) assessed the relationship between overall mitochondrial copy number in blood, and all variables in models 1 and 2. Complete data were available for 953 individuals in Model 3, which had an adjusted R^2^ = 0.084.

Among individuals with asthma, SABA and ICS medication exposure was inversely associated with mitochondrial copy number (i.e., use associated with lower counts–see Supplementary S5 Table); therefore, asthma medication exposure is unlikely to account for differences in copy number between asthma cases and controls. Inverting the models and predicting asthma status did not diminish the statistical significance of the relationship between asthma and mitochondrial copy number in either the SAPPHIRE or SAGE II cohorts (Supplementary S6 and S7 Tables, respectively). Among SAPPHIRE participants with asthma, mitochondrial copy number was not associated with either asthma severity, as measured by time to exacerbation (Supplementary S8 Table), or asthma control (Supplementary S9 Table).

**Fig 2:**
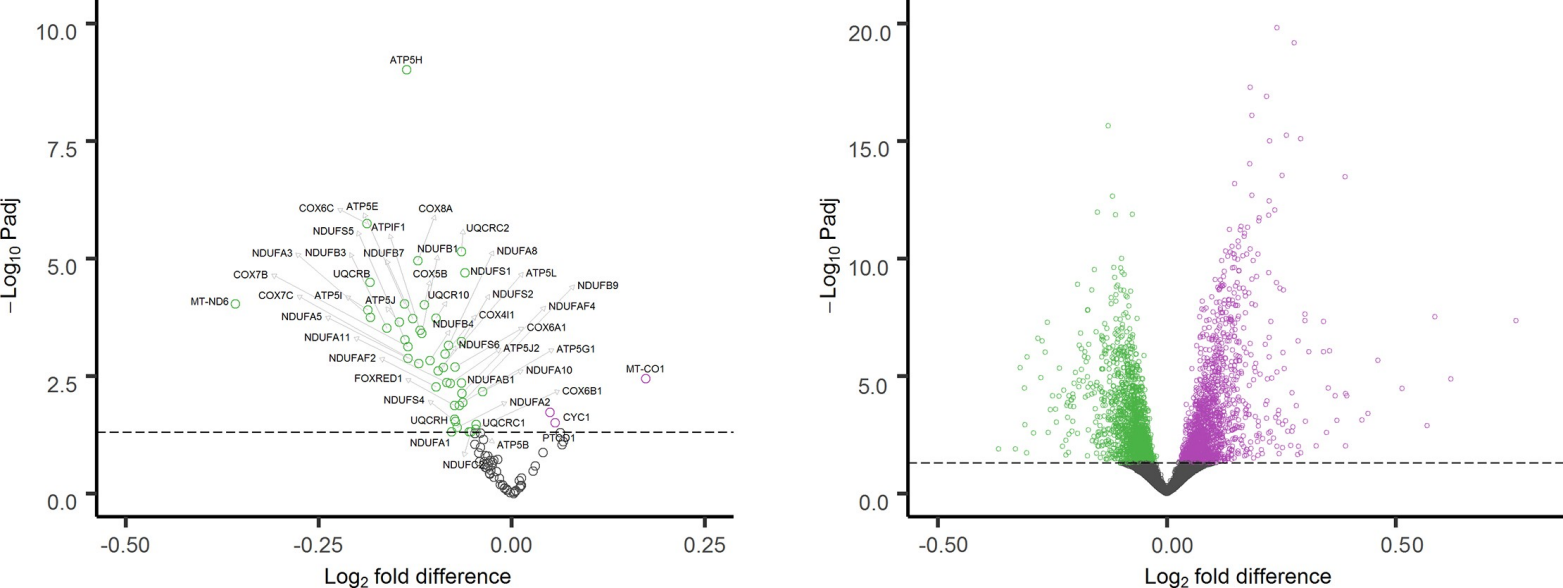
Volcano plots of gene expression differences for SAPPHIRE participants with asthma (n = 197) as compared with participants without asthma (n = 419). Plot A presents expression differences for 100 electron transport chain (ETC) genes derived from RNA-seq of whole blood. Plot B presents expression differences for 18,484 protein-encoding genes not involved in the ETC from the same participant samples. Genes with significantly higher expression in individuals with asthma are shown in purple; genes with significantly lower expression are shown in green; and genes whose expression does not differ significantly between individuals with and without asthma are shown in gray.

RNA-seq data from SAPPHIRE participants with (n = 197) and without (n = 419) asthma were used to evaluate differences in ETC respiratory complex gene expression (Supplementary S10 Table). In the adjusted expression analysis, 48 of 100 respiratory complex genes were significantly (FDR adjusted P-value <0.05) differentially expressed between individuals with and without asthma. Only 3 (*PTCD1*, *CYC1* and *MT-CO1*) of the 48 significantly differentially expressed genes were more highly expressed in individuals with asthma; the other 45 had lower expression in individuals with asthma as compared with individuals without asthma (Fig 2A). In contrast, an evaluation of the other 18,484 protein-encoding genes with >10 total reads across all individuals showed a symmetric distribution for genes with significantly higher (n = 1,627) and significantly lower (n = 1,603) expression for individuals with asthma as compared with those without asthma (Fig 2B). The distribution observed for ETC genes differed significantly from the distribution observed for the other protein-encoding genes (P = 3.97x10^-21^).

Individuals with asthma and high mitochondrial copy number had lower average MitoTracker intensities in blood eosinophils (16,279.8 ± 5,414.7 SD) when compared with controls without asthma (25,693.3 ± 21,181.4 SD), but these differences were not statistically significant (P = 0.525) (Supplementary S11 Table). Individuals with both asthma and an average mitochondrial number had a similar average eosinophil MitoTracker intensity (29,350.5 ± 21,897.1 SD) when compared with controls (P = 0.833). Neutrophils showed the same pattern among individuals with asthma and high mtDNA copy number, asthma with average mitochondrial number, and controls– 13,510.2 (± 5,453.0 SD), 24,581.5 (± 18,482.5 SD), and 23,865.7 (± 21,196.3 SD), respectively. With the exception of monocytes, average MitoTracker intensity was lower in all other cell types assessed (i.e., NK-cells, B-lymphocytes, and T-helper cells) among individuals with asthma and high mitochondrial counts when compared with control individuals.

## Discussion

To our knowledge this is the first study to investigate the association between mitochondrial copy number and asthma status. Specifically, individuals with asthma were observed to have on average higher mitochondrial copy numbers when compared with individuals without asthma. Both cohorts comprised individuals from a similar ancestral background, yet the two study populations were largely non-overlapping with respect to age (i.e., <20 years vs. ≥ 18 years at the time of enrollment) and U.S. locale (i.e., West Coast vs. Midwest). Therefore, the association was robust to these differences, as well as adjustment for multiple potential confounders and explanatory variables. Although leukocytes and platelets are the source of mitochondria in peripheral blood, white cell counts were actually inversely associated with intracellular mtDNA numbers and platelet counts were positively associated with mitochondrial copy numbers; these findings have been reported previously [46]. Mitochondrial copy number appeared to be consistently higher among individuals with asthma at all cell count levels, and the relationship between asthma and copy number persisted after adjustment for leukocyte and platelet numbers. Mitochondrial copy number also appeared to be unrelated to asthma severity or level of control. Together these observations suggest that higher mitochondrial copy numbers in WBCs may be intrinsic to asthma.

Higher mitochondrial copy numbers in peripheral blood leukocytes have been associated with either higher or lower risk for a number of other disease conditions, including development of chronic kidney disease (lower risk) [47], cardiovascular disease (lower risk) [48], diabetes (lower risk and later onset) [49,50], rheumatoid arthritis (lower risk) [51], and multiple malignancies, such as breast cancer (greater risk) [52], chronic lymphocytic leukemia/small lymphocytic lymphoma (greater risk) [53,54], and renal cell carcinoma (lower risk) [55]. Given these multiple associations and the substantial overlap in the distribution of counts between asthma cases and controls, it is unlikely that mitochondrial copy number alone will prove useful as a predictive or diagnostic measure for asthma. Nevertheless, understanding the mechanisms driving the higher mitochondrial counts in individuals with asthma may result in better disease phenotyping and treatment. One twin study has suggested that the heritability of mitochondrial copy number in lymphocytes is 65% [55]. Given the likely polygenic etiology of both mitochondrial copy number and asthma, it is conceivable these two traits share one or more genetic risk factors.

Oxidative stress and the overproduction of damaging ROS are considered features of asthma [56,57]. Inflammatory cells, such as neutrophils, macrophages, and eosinophils, are capable of producing significant increases in ROS when activated [58]. Without sufficient enzymatic and non-enzymatic antioxidant activity, these oxygen radicals can promote cellular apoptosis, damage tissue, and increase airway reactivity [59]. Leukocytes from individuals with asthma have been shown to generate greater levels of superoxide (O_2_^-^) and have lower antioxidant activity when compared with cells from healthy controls [60,61]. Zmijewski and colleagues found that mitochondrial membrane complexes play a central role in regulating ROS levels, and that selective inhibition of complexes I and III could reduce lipopolysaccharide induced lung injury in mice [62,63]. We found that the expression of both nuclear- and mitochondrial-encoded respiratory complex genes were more likely to be lower among individuals with asthma when compared with individuals without asthma. Hence, the lower expression of these genes among individuals with asthma could be a compensatory response to oxidative stress in order to limit inflammatory tissue damage. Oxidative stress can also stimulate an increase in mitochondrial copy number [14]. With diminished ETC function, a proliferative reaction to ROS production could be a compensatory response to maintain cellular energy levels [15].

We also used flow cytometry to identify whether rhodamine-based mitochondrial dye retention in blood leukocytes was influenced by asthma status and mitochondrial copy number. Cellular uptake of rhodamine-based dyes is influenced by mitochondrial number and membrane potential, such that both higher mitochondrial numbers and functioning electron transport with lower proton leak result in greater dye retention [64]. We obtained white blood cells from individuals with asthma and high mtDNA copy numbers, as well as from individuals with and without asthma with average mitochondrial copy numbers. Among cells capable of high ROS production (i.e., eosinophils and neutrophils), MitoTracker staining was lower in individuals with asthma and high mitochondrial copy number when compared with individuals with average mitochondrial copy numbers (with and without asthma). These differences were not statistically different; however, their consistency suggests that rhodamine dye uptake was more profoundly affected by the loss of mitochondrial membrane potential than by the overall increase in mitochondrial copy number. This would be consistent with the observed lower ETC gene expression among participants with asthma, and it suggests that copy number increases are a response to intracellular oxidative stress and reduced ETC function in individuals with asthma. Since a small number of samples were evaluated, these interpretations should be considered highly speculative; nevertheless, these results are useful for generating hypotheses and providing a direction for future investigations.

It is possible that differences in mitochondrial copy number by asthma status reflect environmental exposures. For example, exposure to volatile organic compounds, such as benzene, have been separately associated with higher blood mitochondrial copy numbers and childhood asthma [65,66]. However, the effect of environmental pollutants may differ by exposure type. Exposure to elemental carbon and particulate matter (PM_10_) has been associated with lower mitochondrial copy numbers in the blood of adults [67], but is associated with an increased risk of asthma diagnosis in young children [68]. In our study, we did not observe a significant relationship between smoking status and mitochondrial copy number in blood. However, other studies of individuals exposed to either tobacco smoke or high levels of polycyclic aromatic hydrocarbons suggest that the relationship to mitochondrial copy number is complex and may require better accounting of environmental exposures and individual respiratory complex function than performed here [69,70].

Other study limitations must be considered when evaluating this study. First, since we included only individuals who self-identified as African American in our analysis, our findings may not be generalizable to other populations groups, such as Europeans and Asians. However, as many African Americans have both European and African ancestry [71,72], we assessed and did not find that adjusting for genetic ancestry and mitochondrial haplogroup abrogated the significant relationship between asthma status and mitochondrial copy number. Second, PCR-based quantification is the most common and accepted method to assess mitochondrial DNA copy number [23,73], whereas we used the results of massively parallel WGS to estimate mtDNA copy number. Methods have been developed to rapidly calculate copy number based on WGS data and have been employed in large studies [22,74], but there have been few head-to-head comparisons with PCR-based methods. A recent study using exome sequence data showed moderately good agreement with real time PCR methods for quantifying mitochondrial copy number [75]. In a set of 50 samples, we also showed that PCR-based and sequencing-based assessments of mitochondrial copy number were strongly correlated (albeit different in absolute number). Third, we did not specifically evaluate rhodamine-based mitochondrial dye uptake in platelets. The observed significant interaction between platelet counts and asthma status on mitochondrial copy number suggests that platelets may be a source of some of the higher mitochondrial counts seen in individuals with asthma; this deserves further evaluation. Lastly, we did not directly measure intracellular differences in ROS production or mitochondrial respiration between individuals with and without asthma, and this could be an important next step to further support our observations and hypotheses.

In summary, we performed the first assessment of mitochondrial copy number with asthma status and identified an association that was robust to adjustment for multiple potential explanatory and confounding variables. We further evaluated this relationship in the context of mitochondrial ETC gene expression and rhodamine-based dye uptake by leukocytes. From the resulting observations, we hypothesize that mitochondrial copy number is a manifestation of underlying intracellular oxidative stress in individuals with asthma. Our findings are supported by a number of studies showing that stimulated and unstimulated inflammatory cells (i.e., eosinophils, neutrophils, and monocytes) from individuals with asthma produce more ROS when compared with individuals without asthma [76,77], and also by a study showing that exhaled air condensate from children with stable asthma had higher peroxide levels when compared with healthy controls [78]. Inhaled corticosteroid treatment has been shown to restore antioxidant superoxide dismutase enzyme activity in bronchial epithelial cells from individuals with asthma to levels similar to healthy controls [79], and we found that ICS use among individuals with asthma was significantly and negatively associated with mtDNA copy number. Therefore, mitochondrial copy number may prove to be a subtle sign of ongoing inflammation, a measure of therapeutic response, or an indicator for specific types of treatments (e.g., anti-oxidants) among individuals with asthma; however, this will require additional research.

## Supporting information

S1 Fig **Correlation between mitochondrial DNA (mtDNA) copy number measurements in whole blood using whole genome sequencing (WGS) read depth (y-axis) and real-time PCR quantification (x-axis).** Mitochondrial copy number estimated by WGS is on the y-axis and copy number estimated by real-time PCR quantification is on the x-axis.
(TIF)

S2 Fig Relationship between peripheral blood cell counts and mitochondrial copy number stratified by asthma status. Subfigures show the relationship for neutrophils (**A**), monocytes (**B**), lymphocytes (**C**), eosinophils (**D**), and platelets (**E**). Individuals with asthma are shown in red and individuals without asthma are shown in blue. The interaction P-value is derived from the linear adjusted model for the differences in the relationships by asthma status. The shaded areas represent the 95% confidence interval.
(TIF)

S1 Table Evaluation of the relationship between mitochondria haplogroup and copy number among African American participants in both the SAPPHIRE and SAGE II cohorts. (DOCX)

S2 Table Evaluation of the relationship between mitochondria haplogroup and copy number among African American participants in the SAPPHIRE and SAGE II cohorts stratified by asthma status. (DOCX)

S3 Table Evaluation of the factors associated with mitochondrial copy number among African American SAPPHIRE participants after excluding individuals with African ancestry >5 standard deviation below average and closer than 3rd degree relationship with other participants. (DOCX)

S4 Table Evaluation of the relationship between both asthma status and peripheral white blood counts on mitochondrial copy number among SAGE II participants. (DOCX)

S5 Table Evaluation of the factors associated with mitochondrial copy number restricted to SAPPHIRE participants with asthma. (DOCX)

S6 Table Evaluation of factors related to asthma status, including mitochondrial copy number, among African American individuals from the SAPPHIRE cohort. (DOCX)

S7 Table Evaluation of factors related to asthma status, including mitochondrial copy number, among African American individuals from the SAGE II cohort. (DOCX)

S8 Table Evaluation of factors related to asthma exacerbation, including mitochondrial copy number, among African American SAPPHIRE participants with asthma. (DOCX)

S9 Table Evaluation of factors related to uncontrolled asthma, including mitochondrial copy number, among African American SAPPHIRE participants with asthma. (DOCX)

S10 Table Differences in gene expression by asthma status for protein-encoding genes involved in the mitochondrial electron transport chain. The analysis was performed in SAPPHIRE participants with and without a diagnosis of asthma.
(DOCX)

S11 Table Average rhodamine-based mitochondrial dye (MitoTracker^TM^, Molecular Probes, Inc., Eugene, OR) retention in peripheral white blood cell populations among individuals with asthma and high mitochondrial copy number counts, individuals with asthma and average mitochondrial counts, and individuals without asthma and average mitochondrial counts. (DOCX)

## Abbreviations

ACT: Asthma Control Test
ETC: electron transport chain
FEV_1_: percent of predicted forced expiratory volume at 1 second
MDI: metered dose inhaler
mtDNA: mitochondrial DNA
ROS: reactive oxygen species
SABA: short-acting beta-agonist
SAPPHIRE: Study of Asthma Phenotypes and Pharmacogenomic Interactions by Race-ethnicity
SAGE II: Study of African Americans, Asthma, Genes & Environment
TOPMed: Trans-Omics for Precision Medicine
WBC: white blood cel
