# Supplementary figures and images for: Asthma and its relationship to mitochondrial copy number: Results from the Asthma Translational Genomics Collaborative (ATGC) of the Trans-Omics for Precision Medicine (TOPMed) program
Source: PLoS One. 2020 Nov 25;15(11):e0242364. doi: 10.1371/journal.pone.0242364 (PMC7688161; doi:10.1371/journal.pone.0242364)

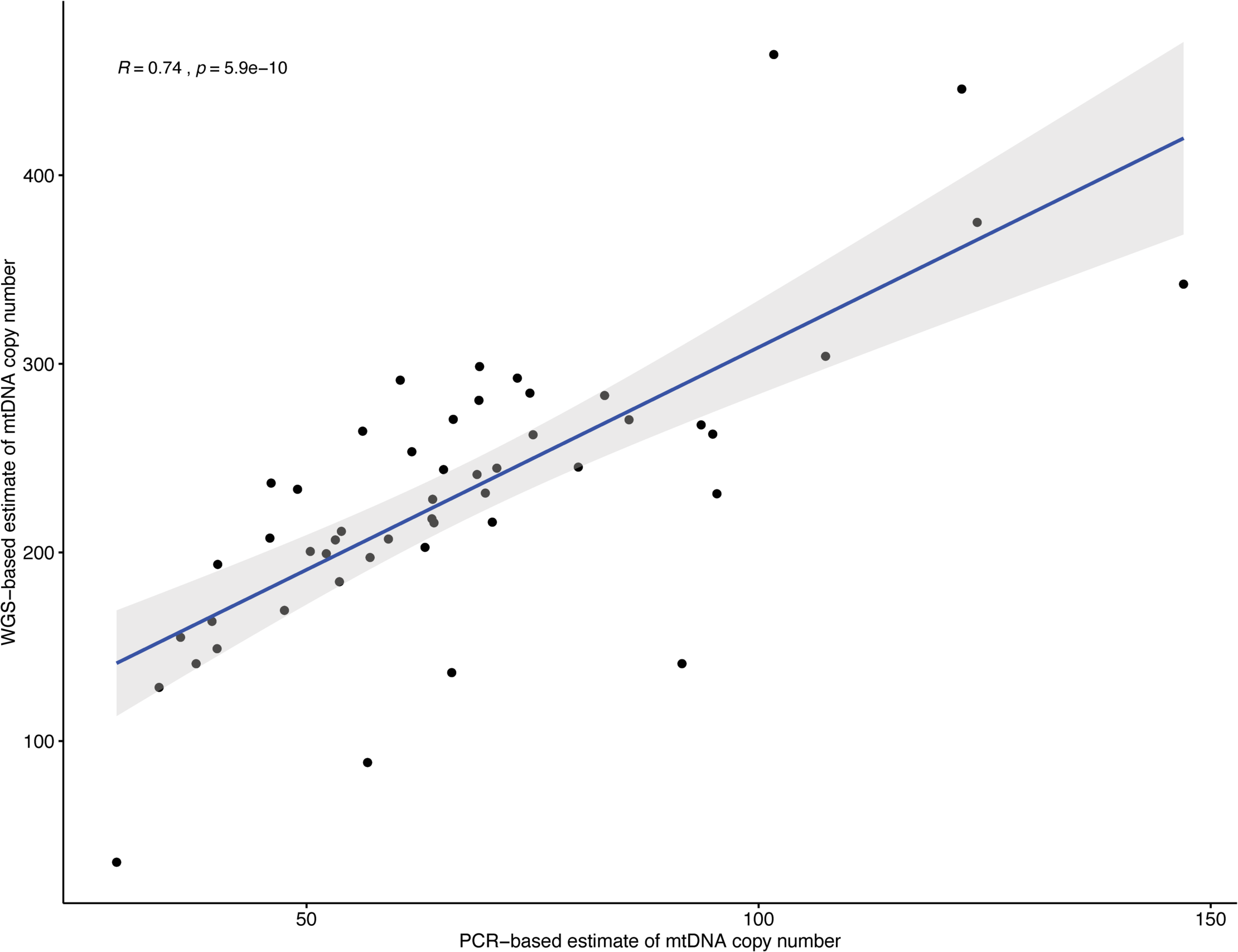

Supplement: S1 Fig — Correlation between mitochondrial DNA (mtDNA) copy number measurements in whole blood using whole genome sequencing (WGS) read depth (y-axis) and real-time PCR quantification (x-axis). Mitochondrial copy number estimated by WGS is on the y-axis and copy number estimated by real-time PCR quantification is on the x-axis. (TIF) [file pone.0242364.s001.tif]

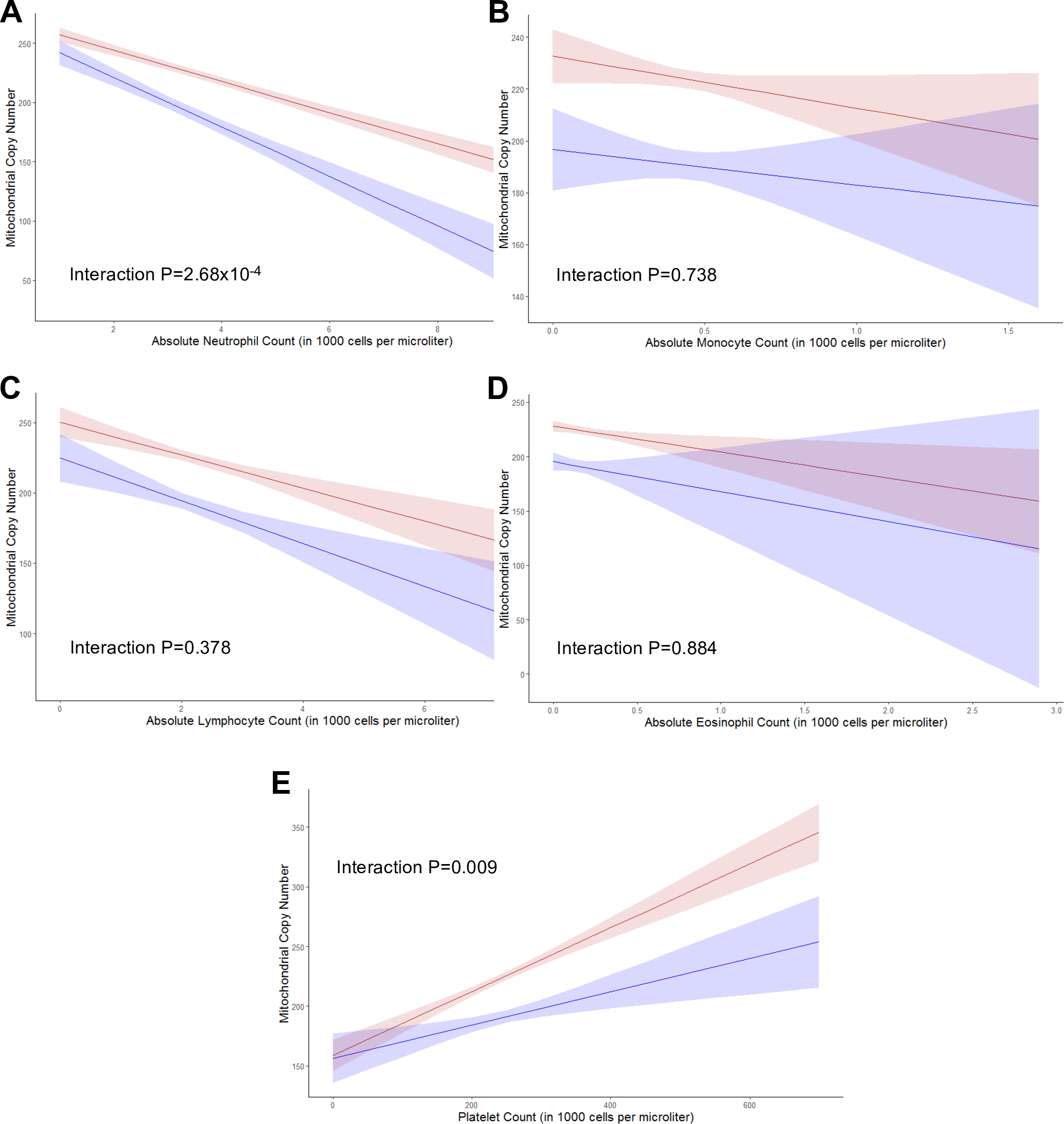

Supplement: S2 Fig — Subfigures show the relationship for neutrophils (A), monocytes (B), lymphocytes (C), eosinophils (D), and platelets (E). Individuals with asthma are shown in red and individuals without asthma are shown in blue. The interaction P-value is derived from the linear adjusted model for the differences in the relationships by asthma status. The shaded areas represent the 95% confidence interval. (TIF) [file pone.0242364.s002.tif]
