# Supplementary material for: Asthma and its relationship to mitochondrial copy number: Results from the Asthma Translational Genomics Collaborative (ATGC) of the Trans-Omics for Precision Medicine (TOPMed) program
Source: PLoS One. 2020 Nov 25;15(11):e0242364. doi: 10.1371/journal.pone.0242364 (PMC7688161; doi:10.1371/journal.pone.0242364)
Supplement: S1 Table — (DOCX) [file pone.0242364.s003.docx]

**S1 Table. Relationship between mitochondria haplogroup and copy number among African American participants in the SAPPHIRE and SAGE II cohorts***

| **Mitochondrial haplogroup†** | **SAPPHIRE cohort** | | | | **SAGE II cohort** | | | |
| --- | --- | --- | --- | --- | --- | --- | --- | --- |
|  | **Number of participants‡** | **African ancestry**  **(mean ± SD)§** | **Copy number (mean ± SD)\|\|** | **P-value¶** | **Number of participants‡** | **African ancestry**  **(mean ± SD)§** | **Copy number (mean ± SD)\|\|** | **P-value¶** |
| L0 | 164 | 0.81 ± 0.09 | 201.95 ± 59.25 | 0.342 | 64 | 0.82 **±** 0.09 | 228.63 **±** 59.02 | 0.407 |
| L1 | 682 | 0.82 ± 0.09 | 215.91 ± 60.95 | 0.047 | 243 | 0.80 **±** 0.11 | 228.75 **±** 52.67 | 0.203 |
| L2 | 1088 | 0.82 ± 0.09 | 211.52 ± 58.99 | 0.309 | 370 | 0.81 **±** 0.10 | 228.66 **±** 60.10 | 0.198 |
| L3 | 1408 | 0.82 ± 0.09 | 218.27 ± 62.84 | 0.006 | 465 | 0.81 **±** 0.09 | 237.46 **±** 65.52 | 0.004 |
| L4 | 29 | 0.81 ± 0.07 | 215.96 ± 75.74 | 0.563 | 7 | 0.87 ± 0.06 | 242.33 ± 51.62 | 0.332 |
| M | 56 | 0.77 ± 0.18 | 222.60 ± 46.38 | 0.037 | 27 | 0.66 ± 0.18 | 232.23 ± 56.58 | 0.365 |
| N+R | 249 | 0.62 ± 0.26 | 207.49 ± 55.68 | Ref | 143 | 0.63 ± 0.18 | 221.34 ± 56.51 | Ref |

SAPPHIRE denotes the Study of Asthma Phenotypes and Pharmacogenomic Interactions by Race-ethnicity; SAGE II, Study of African Americans, Asthma, Genes, & Environment II; and SD, standard deviation.

*The SAPPHIRE study sample was restricted to participants aged ≥18 years at enrollment and the SAGE II study samples was restricted to participants aged <20 years at enrollment.

†As shown in Figure 1, the M haplogroups consist of D, E, C/Z, M7 and other M; the N macrohaplogroups consist of X, A, W, I, and the R sub-macrohaplogroups; and R sub-macrohaplogroups consist of U/K, B, F, HV/H/V, and J/T.

§African ancestry was estimated using a set of autosomal markers which spanned the nuclear genome.

||The mitochondrial copy number estimate was for whole blood. It was based on the sequencing read depth ratio between mitochondrial and nuclear DNA isolated from blood leukocytes.

¶P-values were calculated using the Welch two sample t-test to compare mitochondrial copy numbers between the combined N + R haplogroups (referent) and the other haplogroups.
