# Supplementary material for: Asthma and its relationship to mitochondrial copy number: Results from the Asthma Translational Genomics Collaborative (ATGC) of the Trans-Omics for Precision Medicine (TOPMed) program
Source: PLoS One. 2020 Nov 25;15(11):e0242364. doi: 10.1371/journal.pone.0242364 (PMC7688161; doi:10.1371/journal.pone.0242364)
Supplement: S2 Table — (DOCX) [file pone.0242364.s004.docx]

**S2 Table. Relationship between mitochondria haplogroup and copy number among African American participants in the SAPPHIRE and SAGE II cohorts stratified by asthma status***

| **Mitochondrial haplogroup†** | **SAPPHIRE cohort** | | | | | | **SAGE II cohort** | | | | | **Meta-analysis** | | |
| --- | --- | --- | --- | --- | --- | --- | --- | --- | --- | --- | --- | --- | --- | --- |
|  | **Participants with asthma** | | **Participants without asthma** | | **P-value§** | **Participants with asthma** | | | **Participants without asthma** | | **P-value§** | **Standardized difference (95% CI)** | **Heterogeneity P-value\|\|** | **P-value¶** |
|  | **No.** | **Mitochondria copy number (mean ± SD)‡** | **No.** | **Mitochondria copy number (mean ± SD)‡** |  | **No.** | | **Mitochondria copy number (mean ± SD)‡** | **No.** | **Mitochondria copy number (mean ± SD)‡** |  |  |  |  |
| L0 | 122 | 204.66 ± 53.87 | 42 | 194.09 ± 72.85 | 0.392 | 42 | | 232.17 **±** 49.65 | 22 | 221.87 **±** 74.60 | 0.564 | 0.18 (-0.11-0.47) | 0.988 | 0.233 |
| L1 | 516 | 220.56 ± 60.50 | 166 | 201.44 ± 60.22 | <0.001 | 149 | | 232.97 **±** 48.13 | 94 | 222.06 **±** 58.82 | 0.133 | 0.28 (0.14-0.43) | 0.496 | <0.001 |
| L2 | 826 | 216.40 ± 56.96 | 262 | 196.14 ± 62.65 | <0.001 | 225 | | 237.64 **±** 59.24 | 145 | 214.73 **±** 58.95 | <0.001 | 0.36 (0.24-0.48) | 0.754 | <0.001 |
| L3 | 1110 | 221.65 ± 60.33 | 298 | 205.67 ± 70.11 | <0.001 | 291 | | 240.19 **±** 66.28 | 174 | 232.89 **±** 64.14 | 0.242 | 0.21 (0.10-0.32) | 0.214 | <0.001 |
| L4 | 22 | 227.94 ± 80.12 | 7 | 178.30 ± 46.09 | 0.057 | 5 | | 259.88 ± 51.47 | 2 | 198.45 | -- | -- | -- | -- |
| M | 45 | 221.80 ± 44.60 | 11 | 225.87 ± 55.35 | 0.824 | 17 | | 224.27 ± 43.49 | 10 | 245.77 ± 74.58 | 0.421 | -0.20 (-0.71-0.30) | 0.590 | 0.433 |
| N+R | 187 | 212.13 ± 54.50 | 62 | 193.51 ± 57.30 | 0.027 | 94 | | 226.34 ± 58.31 | 49 | 211.75 ± 52.11 | 0.130 | 0.30 (0.08-0.53) | 0.733 | 0.007 |

SAPPHIRE denotes the Study of Asthma Phenotypes and Pharmacogenomic Interactions by Race-ethnicity; SAGE II, Study of African Americans, Asthma, Genes, & Environment II; SD, standard deviation; and CI, confidence interval.

*The SAPPHIRE study sample was restricted to participants aged ≥18 years at enrollment and the SAGE II study samples was restricted to participants aged <20 years at enrollment.

†As shown in Figure 1, the M haplogroups consist of D, E, C/Z, M7 and other M; the N macrohaplogroups consist of X, A, W, I, and the R sub-macrohaplogroups; and R sub-macrohaplogroups consist of U/K, B, F, and J/T.

‡The mitochondrial copy number estimate was for whole blood. It was based on the sequencing read depth ratio between mitochondrial and nuclear DNA isolated from blood leukocytes.

§P-values were calculated using the Welch two sample t-test to compare mitochondrial copy numbers between individuals with and without asthma.

||Assessment of the difference in P-values from the SAPPHIRE and SAGE II cohorts. A P-value<0.05 would signify a statistically significant difference in the P-values between cohorts.

¶Meta-analysis P-value for the standardized difference in mitochondrial copy number between individuals within and without asthma for the SAPPHIRE and SAGE II cohorts combined.
