# Supplementary material for: Asthma and its relationship to mitochondrial copy number: Results from the Asthma Translational Genomics Collaborative (ATGC) of the Trans-Omics for Precision Medicine (TOPMed) program
Source: PLoS One. 2020 Nov 25;15(11):e0242364. doi: 10.1371/journal.pone.0242364 (PMC7688161; doi:10.1371/journal.pone.0242364)
Supplement: S3 Table — (DOCX) [file pone.0242364.s005.docx]

**S3 Table. Factors associated with mitochondrial copy number among African American SAPPHIRE participants after excluding individuals with African ancestry >5 standard deviation below average and closer than 3^rd^ degree relationship with other participants.**

| **Variable** | **Univariable Analysis** | | | **Model 1†** | | **Model 2‡** | | **Model 3§** | | **Model 4\|\|** | |
| --- | --- | --- | --- | --- | --- | --- | --- | --- | --- | --- | --- |
|  | **R^2^*** | **Unadjusted parameter estimate** | **P-value** | **Adjusted parameter estimate** | **P-value** | **Adjusted parameter estimate** | **P-value** | **Adjusted parameter estimate** | **P-value** | **Adjusted parameter estimate** | **P-value** |
| Asthma status | 0.015 | 18.00 | <0.001 | 19.27 | <0.001 | 34.16 | <0.001 | 18.21 | <0.001 | 33.52 | <0.001 |
| Age (years) | 0.003 | -0.29 | <0.001 | -0.19 | 0.033 | -- | -- | -- | -- | -0.17 | 0.171 |
| Female Sex | <0.001 | 0.27 | 0.903 | 1.97 | 0.388 | -- | -- | -- | -- | -6.14 | 0.070 |
| African Ancestry | 0.003 | 35.35 | <0.001 | 40.50 | <0.001 | -- | -- | -- | -- | 12.87 | 0.399 |
| BMI (kg/m^2^) | 0.002 | -0.35 | 0.002 | -0.39 | 0.001 | -- | -- | -- | -- | 0.07 | 0.694 |
| Smoking status | <0.001 | 1.41 | 0.564 | -1.83 | 0.459 | -- | -- | -- | -- | 8.47 | 0.025 |
| Percent of predicted FEV_1_ | <0.001 | -0.01 | 0.871 | 0.072 | 0.208 | -- | -- | -- | -- | -0.063 | 0.448 |
| Total WBC count | 0.133 | -11.62 | <0.001 | -- | -- | -- | -- | -- | -- | -- | -- |
| Neutrophils | 0.122 | -13.63 | <0.001 | -- | -- | -14.48 | <0.001 | -- | -- | -14.32 | <0.001 |
| Monocytes | 0.043 | -86.59 | <0.001 | -- | -- | -14.39 | 0.142 | -- | -- | -19.26 | 0.060 |
| Lymphocytes | 0.032 | -16.34 | <0.001 | -- | -- | -12.98 | <0.001 | -- | -- | -12.68 | <0.001 |
| Eosinophils | 0.002 | -21.70 | 0.017 | -- | -- | -19.54 | 0.020 | -- | -- | -23.03 | 0.008 |
| Platelet count | 0.007 | 0.10 | <0.001 | -- | -- | 0.214 | <0.001 | -- | -- | 0.23 | <0.001 |
| Mitochondrial haplogroup | 0.004 | -- | -- | -- | -- | -- | -- | -- | -- | -- | -- |
| L0 vs West Eurasian | -- | -2.82 | 0.688 | -- | -- | -- | -- | -2.42 | 0.728 | -2.82 | 0.769 |
| L1 vs West Eurasian | -- | 11.54 | 0.037 | -- | -- | -- | -- | 11.95 | 0.030 | 9.13 | 0.248 |
| L2 vs West Eurasian | -- | 7.37 | 0.168 | -- | -- | -- | -- | 7.66 | 0.149 | 6.53 | 0.393 |
| L3 vs West Eurasian | -- | 14.01 | 0.008 | -- | -- | -- | -- | 13.84 | 0.008 | 13.03 | 0.085 |

SAPPHIRE denotes Study of Asthma Phenotypes and Pharmacogenomic Interactions by Race-ethnicity; BMI, body mass index; FEV_1_, forced expiratory volume at 1 second; and WBC, white blood count.

*The coefficient of determination (R^2^) represented the percent of the variation in the outcome variable (i.e., mitochondrial copy number) that could be accounted for by each variable in the univariable analyses.

†Model 1 assessed the relationship between mitochondrial copy number in blood leukocytes (dependent variable) and asthma status (main explanatory variable). This model adjusted for patient age in years, sex (female=1, male=0), proportion of African ancestry, BMI, smoking status (past or never smoker=0, active smoker=1), and percent of predicted FEV_1_. Complete data were available for 3,449 individuals in Model 1, which had an adjusted R^2^ = 0.024.

‡Model 2 assessed the relationship between mitochondrial copy number in blood leukocytes (dependent variable) and asthma status (main explanatory variable). This model adjusted for absolute white blood cell counts and platelet counts (in increments of 1000 cells per microliter). Complete data were available for 1,915 individuals in Model 2, which had an adjusted R^2^ = 0.219.

§Model 3 assessed the relationship between mitochondrial copy number in blood leukocytes (dependent variable) and asthma status (main explanatory variable). This model adjusted for mitochondrial haplogroup, and only individuals with the L0, L1, L2, L3, and West Eurasian haplogroups were included. Complete data were available for 3,307 individuals in Model 3, which had an adjusted R^2^ = 0.020.

||Model 4 assessed the relationship between mitochondrial copy number in blood leukocytes (dependent variable) and asthma status (main explanatory variable); this model included all of the variables from models 1-3. Complete data were available for 1,831 individuals in Model 4, which had an adjusted R^2^ = 0.229.
