# Supplementary material for: Asthma and its relationship to mitochondrial copy number: Results from the Asthma Translational Genomics Collaborative (ATGC) of the Trans-Omics for Precision Medicine (TOPMed) program
Source: PLoS One. 2020 Nov 25;15(11):e0242364. doi: 10.1371/journal.pone.0242364 (PMC7688161; doi:10.1371/journal.pone.0242364)
Supplement: S4 Table — (DOCX) [file pone.0242364.s006.docx]

**S4 Table. Factors associated with mitochondrial copy number among African American SAGE II participants with white blood cell counts**

| **Variable** | **Model 1*** | | **Model 2†** | |
| --- | --- | --- | --- | --- |
|  | **Adjusted parameter estimate** | **P-value** | **Adjusted parameter estimate** | **P-value** |
| Asthma status | 16.44 | 0.004 | 15.75 | 0.031 |
| Age (years) | -- | -- | -2.15 | 0.026 |
| Female sex | -- | -- | -4.23 | 0.536 |
| African ancestry proportion | -- | -- | 43.57 | 0.226 |
| BMI percentile | -- | -- | 0.21 | 0.127 |
| Smoking status | -- | -- | -- | -- |
| Percent of predicted FEV_1_ | -- | -- | 0.02 | 0.952 |
| Total WBC count | -11.12 | <0.001 | -10.78 | <0.001 |
| Mitochondrial haplogroup | -- | -- | -- | -- |
| L0 vs West Eurasian | -- | -- | -6.31 | 0.767 |
| L1 vs West Eurasian | -- | -- | 1.59 | 0.937 |
| L2 vs West Eurasian | -- | -- | 11.19 | 0.541 |
| L3 vs West Eurasian | -- | -- | 9.60 | 0.596 |

SAGE II denotes the Study of African Americans, Asthma, Genes, & Environment II; BMI, body mass index; FEV_1_, forced expiratory volume at 1 second; and WBC, white blood count.

*Model 1 assessed the relationship between overall mitochondrial copy number in blood and both asthma and WBC. White blood cell count is a continuous variable (in increments of 1000 cells/µl). Complete data were available for 192 individuals in Model 1, which had an adjusted R^2^ = 0.272.

†Model 2 assessed the relationship between overall mitochondrial copy number in blood and asthma while also including the following variables: patient age in years, sex (female=1, male=0), proportion of African ancestry per individual (continuous), body mass index (continuous), smoking status (past or never smoker=0, active smoker=1), percent of predicted FEV1 (continuous), WBC count (in increments of 1000 cells/µl), and mitochondrial haplogroups. Haplogroup was included as categorical variable with West Eurasian as reference level. Only cases with haplogroup L0, L1, L2, L3 and West Eurasian were included in the model. Complete data were available for 151 individuals in Model 2, which had an adjusted R^2^ = 0.287.
