# Supplementary material for: Asthma and its relationship to mitochondrial copy number: Results from the Asthma Translational Genomics Collaborative (ATGC) of the Trans-Omics for Precision Medicine (TOPMed) program
Source: PLoS One. 2020 Nov 25;15(11):e0242364. doi: 10.1371/journal.pone.0242364 (PMC7688161; doi:10.1371/journal.pone.0242364)
Supplement: S5 Table — (DOCX) [file pone.0242364.s007.docx]

**S5 Table. Factors associated with mitochondrial copy number among SAPPHIRE participants with asthma**

| **Variable** | **Univariable Analysis** | | | **Model 1**† | | **Model 2**‡ | | **Model 3§** | | **Model 4\|\|** | |
| --- | --- | --- | --- | --- | --- | --- | --- | --- | --- | --- | --- |
|  | **R^2^*** | **Unadjusted parameter estimate** | **P-value** | **Adjusted parameter estimate** | **P-value** | **Adjusted parameter estimate** | **P-value** | **Adjusted parameter estimate** | **P-value** | **Adjusted parameter estimate** | **P-value** |
| Age (years) | 0.001 | -0.20 | 0.027 | -0.14 | 0.146 | -- | -- | -- | -- | -0.01 | 0.982 |
| Female sex | <0.001 | 0.39 | 0.871 | 2.34 | 0.346 | -- | -- | -- | -- | -7.29 | 0.416 |
| African ancestry proportion | 0.006 | 37.59 | <0.001 | 41.03 | <0.001 | -- | -- | -- | -- | 28.49 | 0.389 |
| BMI (kg/m^2^) | 0.003 | -0.38 | 0.001 | -0.39 | 0.002 | -- | -- | -- | -- | -0.13 | 0.797 |
| Smoking status | <0.001 | -1.42 | 0.569 | -1.79 | 0.484 | -- | -- | -- | -- | 4.35 | 0.731 |
| Percent of predicted FEV_1_ | <0.001 | 0.07 | 0.243 | 0.04 | 0.471 | -- | -- | -- | -- | -0.31 | 0.176 |
| Composite ACT score | <0.001 | 0.35 | 0.106 | 0.23 | 0.324 | -- | -- | -- | -- | 1.38 | 0.143 |
| Total WBC count | 0.160 | -11.92 | <0.001 | -- | -- | -- | -- | -- | -- | -- | -- |
| Neutrophils | 0.141 | -13.53 | <0.001 | -- | -- | -13.25 | <0.001 | -- | -- | -9.68 | <0.001 |
| Monocytes | 0.056 | -95.56 | <0.001 | -- | -- | -22.06 | 0.046 | -- | -- | -9.28 | 0.748 |
| Lymphocytes | 0.034 | -16.22 | <0.001 | -- | -- | -13.11 | <0.001 | -- | -- | -12.71 | 0.024 |
| Eosinophils | 0.006 | -30.13 | 0.001 | -- | -- | -19.48 | 0.025 | -- | -- | -50.92 | 0.017 |
| Platelet count | 0.017 | 0.138 | <0.001 | -- | -- | 0.25 | <0.001 | -- | -- | 0.29 | <0.001 |
| Asthma severity score | <0.001 | -1.75 | 0.545 | -- | -- | -- | -- | 2.03 | 0.568 | 3.17 | 0.547 |
| SABA MDI use | <0.001 | -0.94 | 0.515 | -- | -- | -- | -- | -0.60 | 0.726 | 1.63 | 0.578 |
| SABA nebulizer use | 0.018 | -25.80 | 0.002 | -- | -- | -- | -- | -26.38 | 0.001 | -27.00 | 0.002 |
| ICS use | 0.007 | -6.77 | 0.028 | -- | -- | -- | -- | -7.28 | 0.026 | -16.05 | 0.002 |
| Mitochondrial haplogroup | 0.004 | -- | -- | -- | -- | -- | -- | -- | -- | -- | -- |
| L0 vs West Eurasian | -- | -4.33 | 0.553 | -- | -- | -- | -- | -- | -- | -15.95 | 0.415 |
| L1 vs West Eurasian | -- | 11.58 | 0.039 | -- | -- | -- | -- | -- | -- | -3.10 | 0.845 |
| L2 vs West Eurasian | -- | 7.42 | 0.167 | -- | -- | -- | -- | -- | -- | -9.04 | 0.549 |
| L3 vs West Eurasian | -- | 12.67 | 0.016 | -- | -- | -- | -- | -- | -- | 6.89 | 0.648 |

SAPPHIRE denotes Study of Asthma Phenotypes and Pharmacogenomic Interactions by Race-ethnicity; BMI, body mass index; FEV_1_, forced expiratory volume at 1 second; ACT, asthma control test; WBC, white blood count; SABA, short-acting beta-agonist; MDI, metered dose inhaler; and ICS, inhaled corticosteroid.

*The coefficient of determination (R^2^) represents the proportion of variance in the outcome variable (i.e., mitochondrial copy number) explained by the independent variable.

†Model 1 assessed the relationship between mitochondrial copy number in blood leukocytes (dependent variable) and patient age in years, sex (female=1, male=0), proportion of African ancestry, BMI, smoking status (past or never smoker=0, active smoker=1), and percent of predicted FEV_1_, and composite ACT score (ranging from 5-25 with a higher scores denoting better controlled asthma). Complete data were available for 2,801 individuals in Model 1, which had an adjusted R^2^ = 0.010.

‡Model 2 assessed the relationship between mitochondrial copy number in blood leukocytes (dependent variable) and absolute white blood cell counts as well as platelet counts (in increments of 1000 cells per microliter). Complete data were available for 1,406 individuals in Model 2, which had an adjusted R^2^ = 0.214.

§Model 3 assessed the relationship between mitochondrial copy number and measures of asthma severity and medication use. Complete data were available for 512 individuals in Model 3, which had an adjusted R^2^ = 0.022.

||Model 4 assessed the relationship between mitochondrial copy number and all variables in Models 1, 2, and 3. Model 4 also included variables for mitochondrial haplogroup, and only individuals with the L0, L1, L2, L3, and West Eurasian haplogroups were included. Complete data were available for 247 individuals in Model 4, with adjusted R^2^ = 0.2.
