# Supplementary material for: Asthma and its relationship to mitochondrial copy number: Results from the Asthma Translational Genomics Collaborative (ATGC) of the Trans-Omics for Precision Medicine (TOPMed) program
Source: PLoS One. 2020 Nov 25;15(11):e0242364. doi: 10.1371/journal.pone.0242364 (PMC7688161; doi:10.1371/journal.pone.0242364)
Supplement: S6 Table — (DOCX) [file pone.0242364.s008.docx]

**S6 Table. Factors associated with asthma status among adult African American SAPPHIRE participants**

| **Variable** | **Univariable Analysis** | | **Model 1*** | | **Model 2**† | | **Model 3**‡ | | **Model 4§** | |
| --- | --- | --- | --- | --- | --- | --- | --- | --- | --- | --- |
|  | **OR (95% CI)** | **P-value** | **OR (95% CI)** | **P-value** | **OR (95% CI)** | **P-value** | **OR (95% CI)** | **P-value** | **OR (95% CI)** | **P-value** |
| Age (years) | 0.98 (0.98, 0.99) | <0.001 | 0.97 (0.96, 0.98) | <0.001 | -- | -- | -- | -- | 0.98 (0.97,0.99) | <0.001 |
| Female sex | 1.03 (0.87, 1.22) | 0.734 | 1.25 (1.04, 1.50) | 0.016 | -- | -- | -- | -- | 1.46 (1.14,1.89) | 0.003 |
| African ancestry proportion | 0.67 (0.34, 1.29) | 0.242 | 0.22 (0.11, 0.44) | <0.001 | -- | -- | -- | -- | 0.23 (0.08,0.66) | 0.007 |
| BMI (kg/m^2^) | 1.02 (1.01, 1.03) | <0.001 | 1.02 (1.01, 1.03) | <0.001 | -- | -- | -- | -- | 1.02 (1.01,1.03) | 0.008 |
| Smoking status | 2.57 (2.07, 3.21) | <0.001 | 2.63 (2.09, 3.33) | <0.001 | -- | -- | -- | -- | 2.22 (1.63,3.08) | <0.001 |
| Percent of predicted FEV_1_ | 0.97 (0.96, 0.97) | <0.001 | 0.96 (0.96, 0.97) | <0.001 | -- | -- | -- | -- | 0.97 (0.96,0.97) | <0.001 |
| Absolute WBC counts | -- | -- | -- | -- | -- | -- | -- | -- | -- | -- |
| Neutrophils | 1.19 (1.12, 1.27) | <0.001 | -- | -- | 1.40 (1.29,1.53) | <0.001 | -- | -- | 1.28 (1.17,1.41) | <0.001 |
| Monocytes | 2.20 (1.24, 3.96) | 0.008 | -- | -- | 0.74 (0.36,1.54) | 0.423 | -- | -- | 0.71 (0.32,1.59) | 0.409 |
| Lymphocytes | 1.03 (0.91, 1.16) | 0.646 | -- | -- | 1.04 (0.90,1.20) | 0.584 | -- | -- | 1.07 (0.91,1.25) | 0.417 |
| Eosinophils | 32.08 (14.65, 72.97) | <0.001 | -- | -- | 36.67 (15.91,88.06) | <0.001 | -- | -- | 21.46 (8.81,54.55) | <0.001 |
| Platelet count | 1.00 (1.00,1.00) | 0.986 | -- | -- | 1.00 (1.00,1.00) | 0.001 | -- | -- | 1.00 (0.99,1.00) | <0.001 |
| Mitochondrial copy number | 1.05 (1.04, 1.07) | <0.001 | 1.06 (1.04, 1.07) | <0.001 | 1.09 (1.08,1.11) | <0.001 | 1.05 (1.04, 1.07) | <0.001 | 1.09 (1.07,1.11) | <0.001 |
| Mitochondrial haplogroup | -- | -- | -- | -- | -- | -- | -- | -- | -- | -- |
| L0 vs West Eurasian | 0.82 (0.40, 1.35) | 0.445 | -- | -- | -- | -- | 0.83 (0.50, 1.37) | 0.461 | 1.01 (0.49,2.04) | 0.984 |
| L1 vs West Eurasian | 0.88 (0.59, 1.30) | 0.530 | -- | -- | -- | -- | 0.83 (0.55, 1.22) | 0.352 | 0.82 (0.45,1.47) | 0.513 |
| L2 vs West Eurasian | 0.89 (0.61, 1.29) | 0.562 | -- | -- | -- | -- | 0.86 (0.58, 1.24) | 0.430 | 1.10 (0.61,1.94) | 0.741 |
| L3 vs West Eurasian | 1.06 (0.72,1.52) | 0.772 | -- | -- | -- | -- | 0.99 (0.67, 1.42) | 0.940 | 1.13 (0.63,1.98) | 0.685 |

SAPPHIRE denotes Study of Asthma Phenotypes and Pharmacogenomic Interactions by Race-ethnicity; OR, odds ratio; CI, confidence interval; BMI, body mass index; FEV_1_, forced expiratory volume at 1 second; and WBC, white blood count.

*Model 1 assessed the relationship between asthma status (dependent variable) and mitochondrial copy number (main explanatory variable) per 10 copy increase. This model included variables for patient age in years, sex (female=1, male=0), proportion of African ancestry, BMI, smoking status (past or never smoker=0 vs. active smoker=1), and percent of predicted FEV_1_. Complete data were available for 3675 individuals in Model 1, which had a pseudo R^2^=0.123.

†Model 2 assessed the relationship between asthma status (dependent variable) and mitochondrial copy number (main explanatory variable) per 10 copy increase. This model included variables for absolute white blood cell counts and platelet counts (in increments of 1000 cells/µl). Complete data were available for 2031 individuals in Model 2, which had pseudo R^2^=0.430.

‡Model 3 assessed the relationship between asthma status (dependent variable) and mitochondrial copy number (main explanatory variable) per 10 copy increase. This model included variables for mitochondrial haplogroup, and only individuals with the L0, L1, L2, L3, and West Eurasian haplogroups were included. Complete data were available for 3523 individuals in Model 3, which had a pseudo R^2^=0.016.

§Model 4 assessed the relationship between asthma status (dependent variable) and mitochondrial copy number (main explanatory variable) per 10 copy increase. This model included all of the variables from Models 1-3. Complete data were available for 1942 individuals in Model 4, which had a pseudo R^2^=0.474.
