# Supplementary material for: Asthma and its relationship to mitochondrial copy number: Results from the Asthma Translational Genomics Collaborative (ATGC) of the Trans-Omics for Precision Medicine (TOPMed) program
Source: PLoS One. 2020 Nov 25;15(11):e0242364. doi: 10.1371/journal.pone.0242364 (PMC7688161; doi:10.1371/journal.pone.0242364)
Supplement: S7 Table — (DOCX) [file pone.0242364.s009.docx]

**S7 Table. Factors associated with asthma status among African American SAGE II participants**

| **Variable** | **Univariable Analysis** | | **Multivariable Analysis - Model 1*** | | **Multivariable Analysis - Model 2**† | | **Multivariable Analysis - Model 3**‡ | | **Multivariable Analysis – Model 4§** | | **Multivariable Analysis - Model 5\|\|** | |
| --- | --- | --- | --- | --- | --- | --- | --- | --- | --- | --- | --- | --- |
|  | **OR (95% CI)** | **P-value** | **OR (95% CI)** | **P-value** | **OR (95% CI)** | **P-value** | **OR (95% CI)** | **P-value** | **OR (95% CI)** | **P-value** | **OR (95% CI)** | **P-value** |
| Age (years) | 0.88 (0.85, 0.91) | <0.001 | 0.88 (0.84, 0.92) | <0.001 | -- | -- | -- | -- | 0.87 (0.83, 0.92) | <0.001 | 0.91 (0.81, 1.01) | 0.091 |
| Female sex | 0.73 (0.58, 0.91) | 0.006 | 0.92 (0.68, 1.24) | 0.575 | -- | -- | -- | -- | 0.90 (0.65, 1.23) | 0.494 | 0.48 (0.22, 1.02) | 0.060 |
| African ancestry proportion | 1.41 (0.59, 3.36) | 0.436 | 0.38 (0.11, 1.30) | 0.131 | -- | -- | -- | -- | 0.43 (0.09, 1.95) | 0.280 | 0.19 (0.00, 12.58) | 0.444 |
| BMI percentile | 1.01 (1.00, 1.01) | 0.003 | 1.01 (1.00, 1.01) | 0.002 | -- | -- | -- | -- | 1.01 (1.00, 1.02) | 0.001 | 1.01 (0.99, 1.02) | 0.487 |
| Smoking status | 0.30 (0.01, 3.15) | 0.327 | 1.50 (0.04, 57.18) | 0.816 | -- | -- | -- | -- | 1.50 (0.03, 65.87) | 0.829 | -- | -- |
| Percent of predicted FEV_1_ | 0.98 (0.97, 0.99) | <0.001 | 0.97 (0.96, 0.99) | <0.001 | -- | -- | -- | -- | 0.97 (0.96, 0.99) | <0.001 | 0.95 (0.92, 0.98) | 0.002 |
| Total WBC count | 1.00 (0.87, 1.16) | 0.950 | -- | -- | 1.14 (0.97, 1.36) | 0.129 | -- | -- | -- | -- | 1.10 (0.88, 1.38) | 0.401 |
| Mitochondrial copy number  (per 10 copy increase) | 1.04 (1.02, 1.06) | <0.001 | 1.11 (1.08, 1.14) | <0.001 | 1.12 (1.04, 1.23) | 0.005 | 1.04 (1.02, 1.06) | <0.001 | 1.11 (1.07, 1.14) | <0.001 | 1.13 (1.02, 1.26) | 0.031 |
| Mitochondrial haplogroup | -- | -- | -- | -- | -- | -- | -- | -- | -- | -- | -- | -- |
| L0 vs W.Eurasian | 0.85 (0.43, 1.69) | 0.631 | -- | -- | -- | -- | 0.84 (0.42, 1.67) | 0.610 | 0.86 (0.31, 2.41) | 0.768 | 2.40 (0.22, 26.36) | 0.463 |
| L1 vs W.Eurasian | 0.70 (0.41, 1.17) | 0.183 | -- | -- | -- | -- | 0.69 (0.41, 1.16) | 0.167 | 0.86 (0.37, 1.91) | 0.715 | 14.52 (1.40, 171.39) | 0.026 |
| L2 vs W.Eurasian | 0.69 (0.41, 1.12) | 0.140 | -- | -- | -- | -- | 0.68 (0.41, 1.11) | 0.128 | 0.58 (0.26,1.24) | 0.174 | 4.24 (0.54, 33.96) | 0.160 |
| L3 vs W.Eurasian | 0.74 (0.45, 1.20) | 0.229 | -- | -- | -- | -- | 0.71 (0.43, 1.15) | 0.172 | 0.67 (0.30,1.42) | 0.316 | 2.60 (0.34, 20.02) | 0.344 |

SAGE II denotes the Study of African Americans, Asthma, Genes, & Environment II; OR, odds ratio; CI, confidence interval; BMI, body mass index; FEV_1_, forced expiratory volume at 1 second; and WBC, white blood count.

*Multivariable logistic regression model 1 (Model 1) assessed the relationship between asthma and age in years, sex (female=1, male=0), proportion of African ancestry per individual (continuous), body mass index (continuous), smoking status (past or never smoker=0, active smoker=1), percent of predicted FEV1 (continuous) and mitochondrial copy number (in 10 scale). Complete data were available for 1017 individuals in Model 1, which had AUC=0.717.

†Multivariable logistic regression model 2 (Model 2) assessed the relationship between asthma and individual white blood cell counts as well as mitochondrial copy number (in 10 scale). White blood cell counts were continuous variables (in increments of 1000 cells/µl). Complete data were available for 192 individuals in Model 2, which had AUC = 0.629.

‡Multivariable logistic regression model 3 (Model 3) assessed the relationship between asthma and mitochondrial copy number (in 10 scale) in blood as well as mitochondrial haplogroups (categorical). Complete data were available for 1230 individuals in Model 3, which had AUC = 0.576. Only cases with haplogroup L0, L1, L2, L3 and West Eurasian were included in the model.

§Multivariable logistic regression model 4 (Model 4) included all of the variables from both Model 1 and 3. Complete data were available for 952 individuals in Model 4, which had AUC = 0.726.

||Multivariable logistic regression model 5 (Model 5) included all of the variables from both Model 1, 2 and 3. Complete data were available for 151 individuals in Model 5, which had AUC = 0.771.
