# Supplementary material for: Asthma and its relationship to mitochondrial copy number: Results from the Asthma Translational Genomics Collaborative (ATGC) of the Trans-Omics for Precision Medicine (TOPMed) program
Source: PLoS One. 2020 Nov 25;15(11):e0242364. doi: 10.1371/journal.pone.0242364 (PMC7688161; doi:10.1371/journal.pone.0242364)
Supplement: S8 Table — (DOCX) [file pone.0242364.s010.docx]

**S8 Table.** **Factors associated with asthma exacerbations among SAPPHIRE participants with asthma***

| **Variable** | **Univariable Analysis** | | **Model 1**† | | **Model 2**‡ | | **Model 3**§ | | **Model 4\|\|** | | **Model 5¶** | |
| --- | --- | --- | --- | --- | --- | --- | --- | --- | --- | --- | --- | --- |
|  | **HR**  **(95% CI)** | **P-value** | **HR**  **(95% CI)** | **P-value** | **HR**  **(95% CI)** | **P-value** | **HR**  **95% CI)** | **P-value** | **HR**  **(95% CI)** | **P-value** | **HR**  **(95% CI)** | **P-value** |
| Age (years) | 1.01 (1.00,1.01) | 0.036 | 1.00 (0.99,1.00) | 0.244 | -- | -- | -- | -- | -- | -- | 1.01 (0.99,1.02) | 0.496 |
| Female sex | 1.18 (1.04,1.35) | 0.011 | 1.19 (1.04,1.36) | 0.012 | -- | -- | -- | -- | -- | -- | 1.49 (0.90,2.47) | 0.119 |
| African ancestry | 1.59 (0.95,2.65) | 0.079 | 1.04 (0.62,1.74) | 0.894 | -- | -- | -- | -- | -- | -- | 0.72 (0.18,2.92) | 0.644 |
| BMI (kg/m^2^) | 1.01 (1.00,1.01) | 0.060 | 1.00 (0.99,1.01) | 0.884 | -- | -- | -- | -- | -- | -- | 1.02 (1.00,1.05) | 0.075 |
| Smoking status | 1.13 (0.99,1.28) | 0.070 | 0.95 (0.83,1.09) | 0.467 | -- | -- | -- | -- | -- | -- | 0.69 (0.36,1.34) | 0.270 |
| Percent of predicted FEV_1_ | 0.98 (0.98,0.99) | <0.001 | 0.99 (0.98,0.99) | <0.001 | -- | -- | -- | -- | -- | -- | 0.99 (0.98,1.01) | 0.314 |
| Composite ACT score | 0.94 (0.93,0.95) | <0.001 | 0.95 (0.94,0.97) | <0.001 | -- | -- | -- | -- | -- | -- | 0.93 (0.89,0.98) | 0.003 |
| Total WBC count | 1.05 (1.02,1.09) | 0.003 | -- | -- | -- | -- | -- | -- | -- | -- | -- | -- |
| Neutrophils | 1.08 (1.04,1.12) | <0.001 | -- | -- | 1.09 (1.04,1.14) | <0.001 | -- | -- | -- | -- | 1.07 (0.97,1.19) | 0.179 |
| Monocytes | 1.70 (1.06,2.74) | 0.028 | -- | -- | 1.05 (0.60,1.84) | 0.866 | -- | -- | -- | -- | 0.67 (0.14,3.16) | 0.612 |
| Lymphocytes | 0.91 (0.81,1.01) | 0.074 | -- | -- | 0.85 (0.76,0.95) | 0.005 | -- | -- | -- | -- | 0.97 (0.73,1.28) | 0.807 |
| Eosinophils | 1.99 (1.49,2.67) | <0.001 | -- | -- | 2.04 (1.50,2.77) | <0.001 | -- | -- | -- | -- | 2.35 (0.87,6.37) | 0.093 |
| Asthma severity score | 1.53 (1.34,1.76) | <0.001 | -- | -- | -- | -- | 1.54 (1.31,1.80) | <0.001 | -- | -- | 1.41 (1.12,1.79) | 0.004 |
| SABA MDI use | 1.08 (1.01,1.15) | 0.032 | -- | -- | -- | -- | 0.95 (0.88,1.03) | 0.210 | -- | -- | 0.95 (0.82,1.10) | 0.469 |
| SABA nebulizer use | 1.11 (0.73,1.70) | 0.619 | -- | -- | -- | -- | 1.01 (0.63,1.63) | 0.966 | -- | -- | 1.28 (0.74,2.22) | 0.375 |
| ICS use | 1.30 (1.15,1.46) | <0.001 | -- | -- | -- | -- | 1.18 (1.04,1.35) | 0.013 | -- | -- | 1.28 (0.99,1.64) | 0.056 |
| Mitochondrial copy number | 1.00 (0.99,1.01) | 0.991 | 1.00 (0.99,1.01) | 0.565 | 1.01 (0.99,1.02) | 0.367 | 1.00 (0.97,1.02) | 0.835 | 1.00 (0.99,1.01) | 0.990 | 1.01 (0.98,1.05) | 0.475 |
| Mitochondrial haplogroup | -- | -- | -- | -- | -- | -- | -- | -- | -- | -- | -- | -- |
| L0 vs West Eurasian | 1.20 (0.80,1.78) | 0.377 | -- | -- | -- | -- | -- | -- | 1.20 (0.80,1.78) | 0.377 | 0.91 (0.34,2.42) | 0.843 |
| L1 vs West Eurasian | 1.24 (0.91,1.70) | 0.172 | -- | -- | -- | -- | -- | -- | 1.24 (0.91,1.70) | 0.172 | 0.83 (0.37,1.86) | 0.648 |
| L2 vs West Eurasian | 1.18 (0.87,1.59) | 0.282 | -- | -- | -- | -- | -- | -- | 1.18 (0.87,1.59) | 0.282 | 0.62 (0.28,1.38) | 0.239 |
| L3 vs West Eurasian | 1.17 (0.87,1.57) | 0.309 | -- | -- | -- | -- | -- | -- | 1.17 (0.87,1.57) | 0.309 | 0.65 (0.29,1.46) | 0.297 |

SAPPHIRE denotes Study of Asthma Phenotypes and Pharmacogenomic Interactions by Race-ethnicity; HR, hazard ration; CI, confidence interval; BMI, body mass index; FEV1, forced expiratory volume at 1 second; ACT, asthma control test; WBC, white blood count; SABA, short-acting beta-agonist; MDI, metered dose inhaler; and ICS, inhaled corticosteroid.

*Cox proportional hazards models were used to model the time-to-asthma exacerbation as a function of the variables listed.

Time to event was defined as days from initial visit date to first asthma exacerbation date, censor at most recent visit date after initial visit. Asthma exacerbations were defined as asthma events requiring an oral corticosteroid burst, an emergency room visit, or hospitalization.

†Model 1 accessed the relationship between time-to-asthma exacerbation event (dependent variable) and mitochondrial copy number (main explanatory variable) per 10 copy increase. This model included variables for patient age in years, sex (female=1, male=0), proportion of African ancestry, BMI, smoking status (past or never smoker=0, active smoker=1), percent of predicted FEV1. Complete data were available for 2,450 individuals in Model 1, which had a R2=0.070.

‡Model 2 accessed the relationship between time-to-asthma exacerbation event (dependent variable) and mitochondrial copy number (main explanatory variable) per 10 copy increase. This model included variables for absolute white blood cell counts (in increments of 1000 cells/µl). Complete data were available for 1,250 individuals in Model 2, which had a R2= 0.029.

§Model 3 accessed the relationship between time-to-asthma exacerbation event (dependent variable) and mitochondrial copy number (main explanatory variable) per 10 copy increase. This model included measures of asthma severity and medication use. Complete data were available for 489 individuals in Model 3, which had a R2= 0.079.

||Model 4 accessed the relationship between time-to-asthma exacerbation event (dependent variable) and mitochondrial copy number (main explanatory variable) per 10 copy increase. This model included variables for mitochondrial haplogroup, and only individuals with the L0, L1, L2, L3, and West Eurasian haplogroups were included. Complete data were available for 2,477 individuals in Model 4, which had a R2= 0.001.

¶Model 5 accessed the relationship between time-to-asthma exacerbation event (dependent variable) and mitochondrial copy number (main explanatory variable) per 10 copy increase. This model simultaneously included all of the variables from Models 1, 2, 3 and 4. Complete data were available for 243 individuals in Model 5, which had a R2= 0.219.
