# Supplementary material for: Asthma and its relationship to mitochondrial copy number: Results from the Asthma Translational Genomics Collaborative (ATGC) of the Trans-Omics for Precision Medicine (TOPMed) program
Source: PLoS One. 2020 Nov 25;15(11):e0242364. doi: 10.1371/journal.pone.0242364 (PMC7688161; doi:10.1371/journal.pone.0242364)
Supplement: S9 Table — (DOCX) [file pone.0242364.s011.docx]

**S9 Table. Factors associated with uncontrolled asthma among SAPPHIRE participants with asthma***

| **Variable** | **Univariable Analysis†** | | **Multivariable Analysis‡** | |
| --- | --- | --- | --- | --- |
|  | **OR (95% CI)** | **P-value** | **OR (95% CI)** | **P-value** |
| Age (years) | 1.02 (1.02,1.03) | <0.001 | 1.01 (1.00,1.02) | 0.108 |
| Female Sex | 1.39 (1.18,1.63) | <0.001 | 1.42 (1.09,1.86) | 0.011 |
| African Ancestry | 2.77 (1.50,5.13) | 0.001 | 2.30 (0.79,6.80) | 0.130 |
| BMI (kg/m^2^) | 1.02 (1.01,1.03) | <0.001 | 1.01 (0.99,1.02) | 0.287 |
| Smoking status | 2.64 (2.20,3.17) | <0.001 | 2.79 (2.09,3.75) | <0.001 |
| Percent of predicted FEV_1_ | 0.97 (0.97,0.98) | <0.001 | 0.97 (0.97,0.98) | <0.001 |
| Absolute WBC counts | *--* | *--* | -- | -- |
| Neutrophils | 1.15 (1.08,1.22) | <0.001 | 1.11 (1.03,1.20) | 0.010 |
| Monocytes | 2.44 (1.32,4.56) | 0.005 | 0.85 (0.38,1.91) | 0.700 |
| Lymphocytes | 1.08 (0.94,1.23) | 0.281 | 1.03 (0.88,1.21) | 0.734 |
| Eosinophils | 2.37 (1.34,4.33) | 0.004 | 1.62 (0.86,3.13) | 0.143 |
| Mitochondrial copy number | 0.99 (0.98,1.00) | 0.132 | 1.01 (0.99,1.03) | 0.251 |
| Mitochondrial haplogroup | -- | -- | -- | -- |
| L0 vs W. Eurasian | 1.30 (0.80,2.13) | 0.292 | 0.78 (0.36,1.66) | 0.513 |
| L1 vs W. Eurasian | 1.14 (0.78,1.65) | 0.509 | 0.58 (0.31,1.07) | 0.082 |
| L2 vs W. Eurasian | 1.21 (0.84,1.74) | 0.293 | 0.84 (0.46,1.53) | 0.580 |
| L3 vs W. Eurasian | 1.18 (0.83,1.68) | 0.363 | 0.63 (0.35,1.14) | 0.130 |

SAPPHIRE denotes Study of Asthma Phenotypes and Pharmacogenomic Interactions by Race-ethnicity; OR, odds ratio; CI, confidence interval; BMI, body mass index; FEV_1_, forced expiratory volume at 1 second; ACT, asthma control test; and WBC, white blood count.

*The outcome variable, uncontrolled asthma, was dichotomously defined as a composite ACT score<20 as compared with controlled asthma (composite ACT score ≥20). Therefore, an OR>1 represent an increased risk of uncontrolled asthma.

†Univariable analyses assessed each variable for its relationship to uncontrolled asthma. Categorial variables were defined as follows: sex (female=1, male=0) and smoking status (past or never smoker=0, active smoker=1). Mitochondrial haplogroups L0, L1, L2, L3 were assessed individually in comparison with West Eurasian haplogroups. Due to their small number East Eurasian haplogroups were not included. The remaining variables (age, BMI, absolute WBCs, and mitochondrial copy number) were assessed as continuous variables. Odds ratios for WBCs represent an incremental increase of 1000 cells/µl. Odds ratios for mitochondrial copy numbers represent an incremental increase of 10 copies per cell.

‡The multivariable analysis included all variables simultaneously. Complete data were available for 1,361 individuals; the logistic model had a pseudo R^2^=0.546.
