# Supplementary material for: Asthma and its relationship to mitochondrial copy number: Results from the Asthma Translational Genomics Collaborative (ATGC) of the Trans-Omics for Precision Medicine (TOPMed) program
Source: PLoS One. 2020 Nov 25;15(11):e0242364. doi: 10.1371/journal.pone.0242364 (PMC7688161; doi:10.1371/journal.pone.0242364)
Supplement: S10 Table — The analysis was performed in SAPPHIRE participants with and without a diagnosis of asthma. (DOCX) [file pone.0242364.s012.docx]

**S10 Table. Expression differences by asthma status of genes encoding proteins involved in the mitochondrial electron transport chain.***

| **_Gene ID_** | **_Gene Name_** | **_Genome Location_** | **_Complex_** | **_Asthma Cases_** | | | | **_Controls_** | | | | **_Mann-Whitney P-value†_** | **_Adjusted DESeq2 analysis‡_** | |
| --- | --- | --- | --- | --- | --- | --- | --- | --- | --- | --- | --- | --- | --- | --- |
|  |  |  |  | **_TPM median_** | **_TPM IQR_** | **_TPM mean_** | **_TPM SD_** | **_Control TPM median_** | **_Control TPM IQR_** | **_Control TPM mean_** | **_Control TPM SD_** |  | **_Asthma – parameter estimate_** | **_Asthma - P-value (FDR adjusted)_** |
| ENSG00000198888 | MT_ND1 | Mitochondrial | Complex 1 | 14.882 | 21.524 | 20.353 | 18.772 | 11.160 | 18.065 | 18.284 | 21.977 | 1.76E-01 | 0.002 | 9.96E-01 |
| ENSG00000198763 | MT_ND2 | Mitochondrial | Complex 1 | 11.235 | 19.375 | 18.082 | 18.751 | 10.359 | 14.624 | 15.606 | 16.202 | 5.02E-01 | 0.028 | 3.35E-01 |
| ENSG00000198840 | MT_ND3 | Mitochondrial | Complex 1 | 51.680 | 155.544 | 145.102 | 193.207 | 67.723 | 165.964 | 135.179 | 164.395 | 5.90E-01 | -0.012 | 6.52E-01 |
| ENSG00000198886 | MT_ND4 | Mitochondrial | Complex 1 | 19.973 | 45.556 | 41.150 | 48.211 | 19.943 | 34.149 | 34.661 | 41.261 | 7.18E-01 | 0.031 | 2.60E-01 |
| ENSG00000212907 | MT_ND4L | Mitochondrial | Complex 1 | 18.676 | 56.483 | 40.358 | 48.789 | 20.352 | 43.626 | 35.817 | 44.378 | 8.86E-01 | 0.010 | 5.38E-01 |
| ENSG00000198786 | MT_ND5 | Mitochondrial | Complex 1 | 6.085 | 12.967 | 12.209 | 13.739 | 5.611 | 10.015 | 10.548 | 14.898 | 2.41E-01 | 0.065 | 9.16E-02 |
| ENSG00000198695 | MT_ND6 | Mitochondrial | Complex 1 | 2.422 | 5.730 | 4.882 | 5.711 | 3.162 | 4.109 | 4.918 | 4.970 | **4.55E-02** | -0.358 | **9.25E-05** |
| ENSG00000177646 | ACAD9 | Nuclear | Complex 1 | 7.492 | 5.519 | 8.257 | 5.037 | 7.174 | 5.052 | 7.760 | 3.808 | 6.98E-01 | -0.006 | 8.19E-01 |
| ENSG00000130159 | ECSIT | Nuclear | Complex 1 | 3.663 | 2.375 | 4.075 | 2.081 | 3.276 | 2.073 | 3.795 | 1.996 | 1.14E-01 | 0.063 | 5.06E-02 |
| ENSG00000110074 | FOXRED1 | Nuclear | Complex 1 | 2.286 | 1.992 | 2.615 | 1.553 | 2.266 | 1.640 | 2.587 | 1.470 | 9.50E-01 | -0.074 | **1.35E-02** |
| ENSG00000125356 | NDUFA1 | Nuclear | Complex 1 | 14.195 | 8.913 | 16.340 | 9.070 | 15.104 | 11.503 | 18.249 | 11.588 | 1.74E-01 | -0.078 | **4.84E-02** |
| ENSG00000130414 | NDUFA10 | Nuclear | Complex 1 | 11.293 | 10.108 | 13.724 | 8.648 | 11.167 | 8.162 | 12.891 | 7.186 | 8.62E-01 | -0.038 | **6.78E-03** |
| ENSG00000174886 | NDUFA11 | Nuclear | Complex 1 | 13.293 | 12.930 | 17.269 | 12.358 | 16.095 | 14.050 | 18.631 | 11.610 | 7.34E-02 | -0.084 | **4.36E-03** |
| ENSG00000184752 | NDUFA12 | Nuclear | Complex 1 | 18.890 | 14.068 | 22.117 | 14.798 | 20.776 | 13.820 | 23.905 | 14.532 | 6.90E-02 | -0.029 | 3.75E-01 |
| ENSG00000186010 | NDUFA13 | Nuclear | Complex 1 | 15.541 | 11.495 | 17.431 | 9.959 | 16.596 | 12.463 | 18.647 | 10.010 | 1.62E-01 | -0.035 | 2.73E-01 |
| ENSG00000131495 | NDUFA2 | Nuclear | Complex 1 | 15.238 | 8.219 | 16.079 | 6.064 | 16.559 | 7.288 | 17.301 | 6.125 | 6.90E-02 | -0.071 | **3.97E-02** |
| ENSG00000170906 | NDUFA3 | Nuclear | Complex 1 | 15.684 | 10.817 | 17.105 | 9.852 | 17.776 | 12.180 | 19.775 | 10.474 | **2.40E-02** | -0.183 | **1.78E-04** |
| ENSG00000128609 | NDUFA5 | Nuclear | Complex 1 | 8.132 | 7.925 | 10.643 | 8.323 | 9.653 | 9.959 | 12.395 | 9.379 | **4.55E-02** | -0.134 | **1.33E-03** |
| ENSG00000184983 | NDUFA6 | Nuclear | Complex 1 | 9.647 | 7.235 | 10.832 | 5.822 | 10.522 | 7.565 | 11.955 | 6.614 | 1.46E-01 | -0.028 | 3.87E-01 |
| ENSG00000267855 | NDUFA7 | Nuclear | Complex 1 | 8.877 | 5.033 | 9.572 | 4.172 | 9.238 | 4.830 | 10.104 | 4.151 | 2.41E-01 | -0.036 | 2.45E-01 |
| ENSG00000119421 | NDUFA8 | Nuclear | Complex 1 | 8.794 | 5.696 | 9.441 | 5.057 | 9.604 | 5.897 | 10.324 | 4.648 | **4.55E-02** | -0.082 | **7.06E-04** |
| ENSG00000139180 | NDUFA9 | Nuclear | Complex 1 | 12.253 | 9.609 | 14.829 | 9.418 | 12.730 | 9.333 | 15.028 | 8.666 | 5.17E-01 | -0.018 | 1.88E-01 |
| ENSG00000004779 | NDUFAB1 | Nuclear | Complex 1 | 12.859 | 9.534 | 14.646 | 8.586 | 14.264 | 8.858 | 16.202 | 8.406 | **4.55E-02** | -0.065 | **4.44E-03** |
| ENSG00000137806 | NDUFAF1 | Nuclear | Complex 1 | 5.197 | 4.161 | 6.250 | 4.407 | 5.452 | 3.830 | 6.370 | 3.707 | 2.80E-01 | -0.031 | 1.59E-01 |
| ENSG00000164182 | NDUFAF2 | Nuclear | Complex 1 | 2.507 | 2.151 | 2.769 | 1.543 | 2.725 | 1.960 | 3.204 | 1.861 | **3.47E-02** | -0.098 | **5.42E-03** |
| ENSG00000178057 | NDUFAF3 | Nuclear | Complex 1 | 10.109 | 6.142 | 10.578 | 4.420 | 10.081 | 4.967 | 10.619 | 4.033 | 8.32E-01 | 0.011 | 6.98E-01 |
| ENSG00000123545 | NDUFAF4 | Nuclear | Complex 1 | 2.993 | 2.673 | 3.655 | 2.526 | 3.420 | 2.600 | 4.137 | 2.656 | **4.55E-02** | -0.064 | **7.46E-03** |
| ENSG00000101247 | NDUFAF5 | Nuclear | Complex 1 | 5.521 | 4.736 | 6.786 | 5.017 | 5.327 | 4.319 | 6.421 | 4.195 | 9.37E-01 | 0.010 | 7.54E-01 |
| ENSG00000156170 | NDUFAF6 | Nuclear | Complex 1 | 3.315 | 3.568 | 4.158 | 3.239 | 3.473 | 3.091 | 4.142 | 2.727 | 4.22E-01 | -0.041 | 1.05E-01 |
| ENSG00000183648 | NDUFB1 | Nuclear | Complex 1 | 16.512 | 11.188 | 18.231 | 9.510 | 17.623 | 12.335 | 20.463 | 11.453 | 7.34E-02 | -0.116 | **3.89E-04** |
| ENSG00000140990 | NDUFB10 | Nuclear | Complex 1 | 14.141 | 8.939 | 15.280 | 6.597 | 14.668 | 7.752 | 15.451 | 6.097 | 6.57E-01 | 0.005 | 8.82E-01 |
| ENSG00000147123 | NDUFB11 | Nuclear | Complex 1 | 12.976 | 7.849 | 14.288 | 6.011 | 14.920 | 7.106 | 15.458 | 5.728 | **4.55E-02** | -0.034 | 1.56E-01 |
| ENSG00000090266 | NDUFB2 | Nuclear | Complex 1 | 20.093 | 12.171 | 22.479 | 10.248 | 22.737 | 12.155 | 25.048 | 11.720 | **4.80E-02** | -0.027 | 3.23E-01 |
| ENSG00000119013 | NDUFB3 | Nuclear | Complex 1 | 8.268 | 6.781 | 10.255 | 7.139 | 9.345 | 7.391 | 11.445 | 8.445 | 1.21E-01 | -0.162 | **2.99E-04** |
| ENSG00000065518 | NDUFB4 | Nuclear | Complex 1 | 20.300 | 16.155 | 22.182 | 11.850 | 21.698 | 13.733 | 24.321 | 11.620 | 5.80E-02 | -0.106 | **1.49E-03** |
| ENSG00000136521 | NDUFB5 | Nuclear | Complex 1 | 11.925 | 9.873 | 14.056 | 8.721 | 12.544 | 7.675 | 14.227 | 7.836 | 4.04E-01 | -0.020 | 3.39E-01 |
| ENSG00000165264 | NDUFB6 | Nuclear | Complex 1 | 10.201 | 6.390 | 11.308 | 5.816 | 10.564 | 5.653 | 11.627 | 5.247 | 4.06E-01 | -0.036 | 2.09E-01 |
| ENSG00000099795 | NDUFB7 | Nuclear | Complex 1 | 8.411 | 10.688 | 12.984 | 11.782 | 10.834 | 11.839 | 14.093 | 11.689 | 1.26E-01 | -0.128 | **1.89E-04** |
| ENSG00000166136 | NDUFB8 | Nuclear | Complex 1 | 30.810 | 21.334 | 33.877 | 16.467 | 33.298 | 17.627 | 35.980 | 14.437 | 8.69E-02 | -0.037 | 7.19E-02 |
| ENSG00000147684 | NDUFB9 | Nuclear | Complex 1 | 25.375 | 19.682 | 28.011 | 14.546 | 27.389 | 17.533 | 30.314 | 12.803 | **4.55E-02** | -0.068 | **1.35E-02** |
| ENSG00000109390 | NDUFC1 | Nuclear | Complex 1 | 9.709 | 7.033 | 10.588 | 6.060 | 10.731 | 6.792 | 11.652 | 5.357 | **4.55E-02** | -0.030 | 2.66E-01 |
| ENSG00000151366 | NDUFC2 | Nuclear | Complex 1 | 7.285 | 4.735 | 8.013 | 4.059 | 8.423 | 5.331 | 9.030 | 4.054 | **3.10E-02** | -0.053 | **4.83E-02** |
| ENSG00000023228 | NDUFS1 | Nuclear | Complex 1 | 9.811 | 8.393 | 11.545 | 8.044 | 9.864 | 7.544 | 11.748 | 7.168 | 4.04E-01 | -0.061 | **2.02E-05** |
| ENSG00000158864 | NDUFS2 | Nuclear | Complex 1 | 20.458 | 15.938 | 23.451 | 14.465 | 20.986 | 13.657 | 24.060 | 12.475 | 3.24E-01 | -0.065 | **5.85E-04** |
| ENSG00000213619 | NDUFS3 | Nuclear | Complex 1 | 9.428 | 6.773 | 10.739 | 5.424 | 10.268 | 6.724 | 11.285 | 5.542 | 3.56E-01 | -0.011 | 6.68E-01 |
| ENSG00000164258 | NDUFS4 | Nuclear | Complex 1 | 6.619 | 5.653 | 7.798 | 4.707 | 7.157 | 4.857 | 8.495 | 5.214 | 2.23E-01 | -0.073 | **2.90E-02** |
| ENSG00000168653 | NDUFS5 | Nuclear | Complex 1 | 15.051 | 11.196 | 17.013 | 9.394 | 16.601 | 12.413 | 20.116 | 12.411 | **3.47E-02** | -0.138 | **5.36E-04** |
| ENSG00000145494 | NDUFS6 | Nuclear | Complex 1 | 8.349 | 5.036 | 8.666 | 3.836 | 8.625 | 4.243 | 9.061 | 3.405 | 2.41E-01 | -0.089 | **2.10E-03** |
| ENSG00000115286 | NDUFS7 | Nuclear | Complex 1 | 2.045 | 2.677 | 2.977 | 2.803 | 1.909 | 2.673 | 2.990 | 2.911 | 9.60E-01 | -0.023 | 4.51E-01 |
| ENSG00000110717 | NDUFS8 | Nuclear | Complex 1 | 6.109 | 4.515 | 6.982 | 4.122 | 6.358 | 4.376 | 7.204 | 3.977 | 4.06E-01 | -0.048 | 9.02E-02 |
| ENSG00000167792 | NDUFV1 | Nuclear | Complex 1 | 12.316 | 10.153 | 13.932 | 7.570 | 12.674 | 8.797 | 13.907 | 6.880 | 8.86E-01 | 0.005 | 8.58E-01 |
| ENSG00000178127 | NDUFV2 | Nuclear | Complex 1 | 28.792 | 23.465 | 32.716 | 18.729 | 29.710 | 17.255 | 33.696 | 17.512 | 3.95E-01 | -0.048 | 5.31E-02 |
| ENSG00000160194 | NDUFV3 | Nuclear | Complex 1 | 9.195 | 7.216 | 10.239 | 5.559 | 8.776 | 5.479 | 10.069 | 4.755 | 8.65E-01 | -0.025 | 2.46E-01 |
| ENSG00000151413 | NUBPL | Nuclear | Complex 1 | 2.369 | 2.108 | 2.960 | 2.155 | 2.540 | 2.072 | 2.934 | 1.781 | 4.12E-01 | -0.030 | 2.22E-01 |
| ENSG00000106246 | PTCD1 | Nuclear | Complex 1 | 1.054 | 0.902 | 1.230 | 0.749 | 1.053 | 0.800 | 1.167 | 0.631 | 8.65E-01 | 0.056 | **3.10E-02** |
| ENSG00000073578 | SDHA | Nuclear | Complex 2 | 22.235 | 18.077 | 23.139 | 13.067 | 21.293 | 15.072 | 22.630 | 11.261 | 9.37E-01 | -0.012 | 6.52E-01 |
| ENSG00000117118 | SDHB | Nuclear | Complex 2 | 29.698 | 20.397 | 33.168 | 16.970 | 30.888 | 16.676 | 33.686 | 14.848 | 4.22E-01 | -0.023 | 1.98E-01 |
| ENSG00000143252 | SDHC | Nuclear | Complex 2 | 6.565 | 5.773 | 7.683 | 4.809 | 6.683 | 4.484 | 7.420 | 3.900 | 9.60E-01 | -0.016 | 4.77E-01 |
| ENSG00000204370 | SDHD | Nuclear | Complex 2 | 43.227 | 34.935 | 48.887 | 29.945 | 42.607 | 29.270 | 49.488 | 26.158 | 4.97E-01 | -0.002 | 9.60E-01 |
| ENSG00000198727 | MT_CYB | Mitochondrial | Complex 3 | 20.866 | 37.992 | 34.021 | 32.848 | 17.091 | 27.465 | 28.353 | 36.926 | **4.55E-02** | 0.003 | 9.23E-01 |
| ENSG00000179091 | CYC1 | Nuclear | Complex 3 | 7.232 | 5.256 | 8.488 | 4.679 | 7.394 | 4.830 | 8.450 | 4.413 | 8.15E-01 | 0.050 | **1.88E-02** |
| ENSG00000184076 | UQCR10 | Nuclear | Complex 3 | 18.728 | 10.255 | 19.345 | 6.984 | 20.792 | 10.034 | 21.773 | 7.344 | **1.58E-02** | -0.098 | **1.86E-04** |
| ENSG00000127540 | UQCR11 | Nuclear | Complex 3 | 17.125 | 6.872 | 17.881 | 6.468 | 17.682 | 6.902 | 18.868 | 7.032 | 1.72E-01 | -0.006 | 8.64E-01 |
| ENSG00000156467 | UQCRB | Nuclear | Complex 3 | 5.729 | 4.448 | 7.242 | 5.708 | 6.128 | 6.152 | 9.112 | 8.761 | 5.80E-02 | -0.184 | **3.23E-05** |
| ENSG00000010256 | UQCRC1 | Nuclear | Complex 3 | 19.243 | 13.738 | 20.905 | 10.450 | 19.744 | 10.665 | 20.980 | 8.945 | 7.21E-01 | -0.046 | **3.41E-02** |
| ENSG00000140740 | UQCRC2 | Nuclear | Complex 3 | 33.997 | 25.564 | 41.403 | 25.965 | 36.400 | 24.826 | 42.492 | 22.984 | 2.80E-01 | -0.065 | **7.15E-06** |
| ENSG00000169021 | UQCRFS1 | Nuclear | Complex 3 | 8.030 | 5.700 | 9.260 | 6.001 | 8.261 | 5.556 | 9.467 | 5.204 | 3.56E-01 | 0.013 | 4.70E-01 |
| ENSG00000173660 | UQCRH | Nuclear | Complex 3 | 23.875 | 17.085 | 27.278 | 15.650 | 25.624 | 18.828 | 31.131 | 18.772 | 6.90E-02 | -0.074 | **2.64E-02** |
| ENSG00000164405 | UQCRQ | Nuclear | Complex 3 | 13.796 | 8.117 | 15.146 | 6.367 | 15.399 | 8.981 | 17.050 | 8.027 | **4.80E-02** | -0.043 | 1.39E-01 |
| ENSG00000198804 | MT_CO1 | Mitochondrial | Complex 4 | 263.145 | 398.173 | 417.499 | 427.000 | 227.842 | 315.330 | 343.922 | 350.179 | 2.41E-01 | 0.174 | **3.60E-03** |
| ENSG00000198712 | MT_CO2 | Mitochondrial | Complex 4 | 99.506 | 240.873 | 196.700 | 219.493 | 110.033 | 189.952 | 172.114 | 180.897 | 8.65E-01 | 0.011 | 6.68E-01 |
| ENSG00000198938 | MT_CO3 | Mitochondrial | Complex 4 | 167.423 | 308.243 | 294.332 | 302.998 | 171.076 | 254.173 | 257.261 | 236.766 | 9.06E-01 | 0.003 | 9.38E-01 |
| ENSG00000131143 | COX4I1 | Nuclear | Complex 4 | 32.444 | 18.088 | 33.873 | 13.580 | 35.309 | 17.247 | 38.391 | 15.793 | **3.10E-02** | -0.086 | **1.08E-03** |
| ENSG00000178741 | COX5A | Nuclear | Complex 4 | 7.392 | 5.801 | 8.205 | 4.420 | 7.214 | 4.429 | 8.469 | 4.728 | 6.66E-01 | -0.009 | 7.74E-01 |
| ENSG00000135940 | COX5B | Nuclear | Complex 4 | 30.331 | 18.247 | 33.411 | 15.394 | 34.414 | 17.664 | 36.833 | 15.925 | **3.47E-02** | -0.113 | **9.65E-05** |
| ENSG00000111775 | COX6A1 | Nuclear | Complex 4 | 52.122 | 26.057 | 54.818 | 20.365 | 56.185 | 25.414 | 59.813 | 21.179 | **4.55E-02** | -0.074 | **2.04E-03** |
| ENSG00000126267 | COX6B1 | Nuclear | Complex 4 | 82.577 | 47.743 | 87.823 | 35.464 | 87.679 | 44.178 | 92.229 | 32.642 | 1.46E-01 | -0.055 | **4.85E-02** |
| ENSG00000164919 | COX6C | Nuclear | Complex 4 | 14.406 | 12.757 | 17.139 | 11.194 | 15.858 | 13.309 | 20.349 | 13.887 | **3.47E-02** | -0.188 | **1.80E-06** |
| ENSG00000115944 | COX7A2L | Nuclear | Complex 4 | 19.988 | 12.307 | 22.559 | 11.708 | 20.998 | 11.447 | 23.426 | 10.334 | 2.19E-01 | -0.015 | 6.44E-01 |
| ENSG00000131174 | COX7B | Nuclear | Complex 4 | 6.186 | 4.992 | 7.261 | 4.669 | 6.635 | 5.089 | 8.045 | 5.114 | 1.21E-01 | -0.135 | **7.50E-04** |
| ENSG00000127184 | COX7C | Nuclear | Complex 4 | 41.052 | 31.575 | 47.468 | 29.349 | 45.691 | 37.897 | 55.071 | 34.971 | 6.08E-02 | -0.121 | **1.71E-03** |
| ENSG00000176340 | COX8A | Nuclear | Complex 4 | 32.079 | 21.112 | 33.071 | 15.950 | 32.473 | 17.048 | 35.782 | 15.664 | 1.12E-01 | -0.121 | **1.10E-05** |
| ENSG00000198899 | MT_ATP6 | Mitochondrial | Complex 5 | 5.819 | 10.562 | 9.667 | 10.459 | 4.777 | 7.619 | 7.812 | 9.145 | 1.74E-01 | 0.041 | 1.34E-01 |
| ENSG00000228253 | MT_ATP8 | Mitochondrial | Complex 5 | 7.641 | 23.923 | 20.689 | 27.920 | 7.508 | 14.058 | 16.002 | 22.553 | 8.82E-01 | 0.012 | 6.68E-01 |
| ENSG00000152234 | ATP5A1 | Nuclear | Complex 5 | 71.338 | 52.844 | 80.287 | 45.907 | 74.344 | 49.670 | 84.864 | 42.315 | 2.41E-01 | -0.025 | 2.20E-01 |
| ENSG00000110955 | ATP5B | Nuclear | Complex 5 | 73.339 | 53.692 | 81.701 | 46.964 | 75.634 | 42.385 | 82.963 | 39.289 | 4.04E-01 | -0.047 | **4.25E-02** |
| ENSG00000165629 | ATP5C1 | Nuclear | Complex 5 | 46.847 | 32.041 | 54.362 | 31.511 | 49.363 | 33.984 | 57.287 | 31.900 | 3.24E-01 | -0.009 | 7.58E-01 |
| ENSG00000099624 | ATP5D | Nuclear | Complex 5 | 1.073 | 1.315 | 1.433 | 1.339 | 0.903 | 0.995 | 1.312 | 1.478 | 2.69E-01 | 0.067 | 7.89E-02 |
| ENSG00000124172 | ATP5E | Nuclear | Complex 5 | 35.922 | 17.478 | 38.773 | 17.170 | 38.950 | 19.872 | 42.245 | 18.095 | 6.29E-02 | -0.139 | **9.25E-05** |
| ENSG00000116459 | ATP5F1 | Nuclear | Complex 5 | 31.278 | 28.886 | 39.218 | 25.611 | 37.493 | 29.675 | 43.997 | 26.768 | **4.55E-02** | -0.041 | 5.16E-02 |
| ENSG00000159199 | ATP5G1 | Nuclear | Complex 5 | 14.181 | 9.227 | 15.789 | 7.660 | 15.987 | 9.029 | 17.144 | 7.086 | **4.55E-02** | -0.063 | **1.16E-02** |
| ENSG00000135390 | ATP5G2 | Nuclear | Complex 5 | 31.043 | 24.535 | 35.306 | 21.184 | 33.551 | 26.162 | 37.895 | 20.162 | 1.46E-01 | -0.041 | 2.09E-01 |
| ENSG00000154518 | ATP5G3 | Nuclear | Complex 5 | 8.901 | 6.318 | 10.475 | 6.815 | 9.578 | 6.546 | 10.470 | 5.161 | 3.48E-01 | -0.027 | 1.98E-01 |
| ENSG00000167863 | ATP5H | Nuclear | Complex 5 | 44.826 | 32.421 | 52.075 | 30.010 | 49.658 | 30.523 | 58.291 | 28.834 | **2.86E-02** | -0.136 | **9.60E-10** |
| ENSG00000169020 | ATP5I | Nuclear | Complex 5 | 15.142 | 8.621 | 16.791 | 7.535 | 17.856 | 9.633 | 19.640 | 9.581 | **7.68E-03** | -0.186 | **1.25E-04** |
| ENSG00000154723 | ATP5J | Nuclear | Complex 5 | 7.994 | 9.215 | 10.500 | 8.082 | 9.421 | 9.105 | 11.714 | 8.167 | **4.55E-02** | -0.146 | **2.26E-04** |
| ENSG00000241468 | ATP5J2 | Nuclear | Complex 5 | 20.283 | 16.784 | 24.039 | 14.123 | 22.740 | 16.139 | 25.777 | 13.675 | 9.56E-02 | -0.080 | **4.53E-03** |
| ENSG00000167283 | ATP5L | Nuclear | Complex 5 | 21.086 | 14.084 | 23.249 | 11.378 | 22.550 | 14.227 | 25.710 | 11.865 | **4.97E-02** | -0.095 | **2.44E-03** |
| ENSG00000241837 | ATP5O | Nuclear | Complex 5 | 19.342 | 14.299 | 22.166 | 13.289 | 20.106 | 13.525 | 23.876 | 14.294 | 2.41E-01 | -0.032 | 2.89E-01 |
| ENSG00000130770 | ATPIF1 | Nuclear | Complex 5 | 11.087 | 7.806 | 12.203 | 6.098 | 12.567 | 7.608 | 13.691 | 6.210 | **3.47E-02** | -0.119 | **3.34E-04** |

SAPPHIRE denotes Study of Asthma Phenotypes and Pharmacogenomic Interactions by Race-ethnicity; TPM, transcripts per kilobase per million reads; IQR, interquartile range, and SD standard deviation.

*The SAPPHIRE study sample was restricted to participants aged ≥18 years at enrollment and included 197 individuals with asthma and 419 individuals without asthma.

†P-value for the comparison of the TPM values between individuals with and without asthma. P-values were adjusted by the false discovery rate.

‡The R-package DESeq2 was also used to assess for difference in gene expression by asthma status while adjusting for patient age in years, sex (female=1, male=0), proportion of African ancestry, absolute white blood cell counts (separately for neutrophils, monocytes, lymphocytes, and eosinophils) and platelet counts, and ten surrogate variables. Surrogate variable were calculated using the method described by Leek (Nucleic Acids Res. 2014 Dec 1;42[21]:e161). P-values were adjusted by the false discovery rate.
