# Supplementary material for: Asthma and its relationship to mitochondrial copy number: Results from the Asthma Translational Genomics Collaborative (ATGC) of the Trans-Omics for Precision Medicine (TOPMed) program
Source: PLoS One. 2020 Nov 25;15(11):e0242364. doi: 10.1371/journal.pone.0242364 (PMC7688161; doi:10.1371/journal.pone.0242364)
Supplement: S11 Table — (DOCX) [file pone.0242364.s013.docx]

**S11 Table. MitoTracker^TM^ intensity in leukocytes obtained from individuals with asthma and high mitochondrial counts, individuals with asthma and average mitochondrial counts, and individuals without asthma and average mitochondrial counts.***

| **Group** | **Mitochondrial count (mean ± SD)** | **Eosinophil intensity (mean ± SD) †** | **Neutrophil intensity (mean ± SD) †** | **Monocyte intensity (mean ± SD)‡** | **NK-cell intensity (mean ± SD)§** | **B-lymphocyte intensity (mean ± SD) §** | **T-helper lymphocyte intensity (mean ± SD)\|\|** |
| --- | --- | --- | --- | --- | --- | --- | --- |
| Asthma cases with high mitochondrial counts (n=4) | 371.3 ± 25.3 | 16,279.8 ± 5,414.7 | 13,510.2 ± 5,453.0 | 1,175.2 ± 1,341.9 | 1,791.0 ± 1,708.3 | 1,153.2 ± 1,344.6 | 295.5 ± 137.9 |
| Asthma cases with average mitochondrial counts (n=4) | 217.9 ± 1.3 | 29,350.5 ± 21,897.1 | 24,581.5 ± 18,482.5 | 1,497.7 ± 1,333.2 | 1,520.7 ± 1,378.5 | 1,510.0 ± 1,357.0 | 1,370.0 ± 1,184.7 |
| Control cases with average mitochondrial counts (n=5) | 198.1 ± 5.7 | 25,693.3 ± 21,181.4 | 23,864.7 ± 21,196.3 | 638.3 ± 525.3 | 1,805.4 ± 3,135.7 | 2,317.8 ± 3,767.0 | 663.3 ± 707.8 |

SD denotes standard deviation and NK, natural killer.

* MitoTracker Red CMXRos (Molecular Probes, Inc., Eugene, OR) was used to stain leukocyte populations. Granulocytes (Eosinophils and Neutrophils) were processed separately from mononuclear cells (monocytes, NK-cells, B-lymphocytes, and T-helper lymphocytes).

†Includes 4 individuals with asthma and high mitochondrial counts, 4 individuals with asthma and average mitochondrial counts, and 3 individuals with average mitochondrial counts

‡ Includes 4 individuals with asthma and high mitochondrial counts, 3 individuals with asthma and average mitochondrial counts, and 4 individuals with average mitochondrial counts

§ Includes 4 individuals with asthma and high mitochondrial counts, 3 individuals with asthma and average mitochondrial counts, and 5 individuals with average mitochondrial counts

|| Includes 2 individuals with asthma and high mitochondrial counts, 3 individuals with asthma and average mitochondrial counts, and 5 individuals with average mitochondrial counts
